# At the core of the interaction: Probing charged side chains in flexible protein regions with simultaneous nuclear magnetic resonance experiments

**DOI:** 10.1002/pro.70533

**Published:** 2026-03-13

**Authors:** Maria Anna Rodella, Marco Schiavina, Maksim Mayzel, Carlotta Cappanni, Rainer Kümmerle, Roberta Pierattelli, Isabella C. Felli

**Affiliations:** ^1^ Department of Chemistry “Ugo Schiff” and Magnetic Resonance Center (CERM) University of Florence Florence Italy; ^2^ Bruker BioSpin AG Fällanden Switzerland

**Keywords:** ^13^C detection, arginine, IDRs, nuclear magnetic resonance, protein‐ligand interaction, SARS‐CoV‐2

## Abstract

Charged amino acid side chains are crucial mediators of biomolecular recognition, but their characterization by nuclear magnetic resonance (NMR) is often hindered by conformational and solvent exchange, particularly for arginine guanidinium groups. We present two complementary ^13^C‐detected NMR strategies that exploit multiple acquisition schemes to simultaneously monitor positively and negatively charged residues. A “NMR by Ordered Acquisition using ^1^H detection (NOAH)”‐based experiment combines C_ζ_N_η_‐HDQC and SC‐CACO experiments, allowing the simultaneous detection of arginine, aspartate, and glutamate side chains resonances. In parallel, the Multiple‐Receiver (MR) strategy integrates CP‐HISQC and C_ζ_N_ε_‐HSQC, allowing full assignment of the arginine guanidinium group. We apply this approach to study the interaction between the SARS‐CoV‐2 nucleocapsid N‐terminal domain and the negatively charged glycosaminoglycan enoxaparin. The experiments provide information about flexible, charged side chains at the protein ligand interface. Together, NOAH and MR approaches provide a powerful framework for the high‐resolution characterization of charged side chains and electrostatically driven interactions.

## 1 INTRODUCTION

Charged amino acid side chains play a central role in numerous biomolecular processes, including molecular recognition (Hodges et al., 2015), signaling (Moens et al., 2019; Tsikas, 2021), catalysis (Hsiao et al., 2018; Pauff et al., 2007), and structural stabilization (Meuzelaar et al., 2016). Their ability to form hydrogen bonds, salt bridges, and ionic interactions makes them key mediators in the binding of ligands, nucleic acids, metal ions, and proteins. Among these, surface‐exposed and highly flexible charged residues are of particular interest, as they frequently serve as initial contact points in transient interactions and dynamic molecular assemblies. However, flexibility and solvent exposure pose substantial challenges for their high‐resolution structural and dynamic characterization.

Arginine residues are particularly significant due to the chemical nature of their guanidinium group, which remains positively charged in a large range of conditions, including human physiological ones, and can form multiple directional hydrogen bonds, salt bridges, and cation‐π interactions. Present in active sites of enzymes, surface exposed arginine residues also play a crucial role in protein‐RNA interactions (Barik et al., 2015; Calnan et al., 1991; Smith et al., 2000) and often modulate the onset of liquid–liquid phase separation (Wang et al., 2018). Despite their functional importance, their structural investigation via nuclear magnetic resonance (NMR) spectroscopy remains challenging, in particular for surface exposed residues.

The guanidinium group of arginine residues is prone to conformational exchange and its protons undergo chemical exchange with surrounding water molecules, leading to severe line broadening and to signal loss in conventional ^1^H‐detected experiments (Nguyen et al., 2019). Consequently, critical information on electrostatic interactions and binding events may be lost when using standard NMR approaches. From this perspective, ^13^C‐detection spectroscopy (Felli & Pierattelli, 2022) can reveal unique insights, especially when combined with strategies tailored to alleviate the problems arising from solvent and conformational exchange resulting in broadening of the resonances (Mackenzie & Hansen, 2017; Yoshimura et al., 2017). In case of guanidinium groups of arginine amino acids, the sensitivity in ^13^C‐detection NMR experiments can be enhanced by utilizing J‐based double cross polarization (J‐CP) scheme, which exploits the water polarization to enhance the starting polarization source of exchangeable nuclear spins (Kim et al., 2023; Lopez et al., 2016). Additionally, the resonance broadening mediated by conformational exchange affecting the terminal part of the guanidinium group is mitigated by exploiting multiple quantum coherences involving the ^15^N^η^ nuclear spins (Chang et al., 2014; Mackenzie & Hansen, 2017). Direct ^13^C detected NMR also permits monitoring the side chains of negatively charged residues such as aspartate and glutamate. Simultaneous monitoring of amino‐acid side chains with positive and negative charges provides a very powerful tool to characterize proteins' electrostatic interactions.

The availability of multiple acquisition strategies has prompted the design of experiments in which more than one NMR spectrum can be acquired within the time required for the longest one (Knödlstorfer et al., 2024; Kupče et al., 2021; Pontoriero et al., 2022; Schiavina et al., 2019; Viegas et al., 2016), providing an ideal framework for developing a general strategy to monitor both positively and negatively charged side chains. Our work is based on NOAH (NMR by Ordered Acquisition using ^1^H detection) (Kupče & Claridge, 2017; Pandey et al., 2025; Yong et al., 2022) and UTOPIA (Schiavina et al., 2019; Viegas et al., 2016) (Unified Time‐OPtimized Interleaved Acquisition) frameworks which enable the simultaneous acquisition of multiple spectra providing complementary information. NOAH supersequences have so far been used for small‐molecule NMR, drastically reducing experimental time by nesting multiple 2D experiments into a single sequence, sequentially preserving proton magnetization for subsequent modules, and using a single receiver (same detected nucleus). Multiple‐Receivers (MR) and UTOPIA methods utilize several different receivers to simultaneously detect different nuclei, enabling parallel acquisition of complementary experiments, and are ideal for time‐sensitive protein studies. The experimental approach presented here exploits these two different strategies for the simultaneous acquisition of multiple NMR spectra. Combining two distinct experiments within the timeframe of a single experiment saves time while maintaining the same performance as individual acquisitions and providing complementary perspectives on the studied system.

**FIGURE 1:**
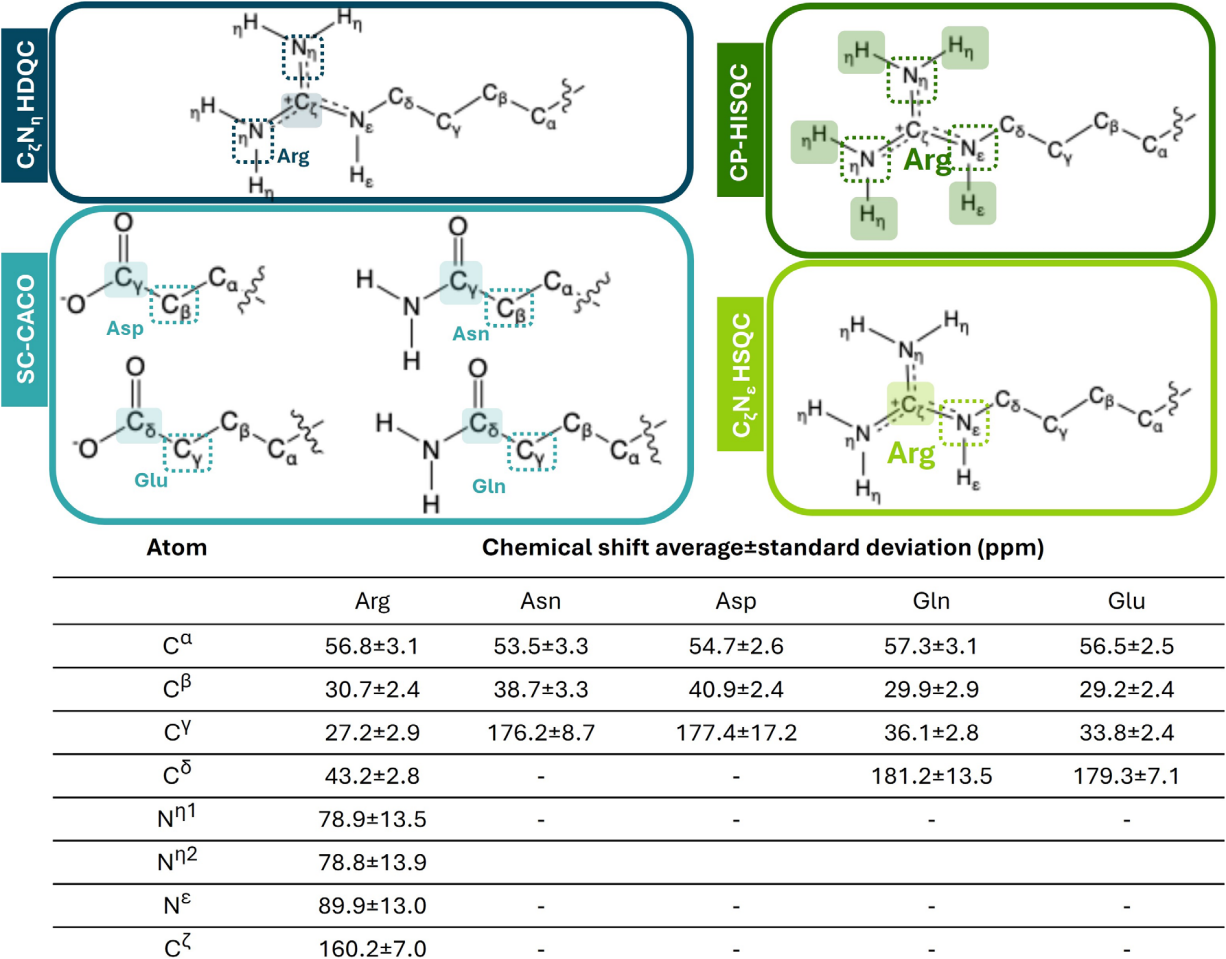
A schematic representation of arginine, glutamate, aspartate, glutamine and asparagine side chains, together with the standard nomenclature used to indicate the nuclei present within each side chains. Amino acids investigated in each experiment are enclosed within rectangles with solid outlines (blue for C_ζ_N_η_ HDQC, light blue for SC‐CACO, dark green for CP‐HISQC, and light green for C_ζ_N_ε_ HSQC). Nuclei directly detected in the experiment are highlighted by filled rectangles, whereas nuclei evolved in the indirect dimension are indicated by rectangles with dashed outlines. A table summarizing the average chemical shifts (in ppm) and their standard deviations for the studied nuclei is also shown; values were obtained from the BMRB (https://bmrb.io).

We propose a new NOAH‐based super‐sequence that employs ^13^C rather than ^1^H detection. As with other NOAH super‐sequences, care must be taken to preserve magnetization transfer between the individual sub‐experiments. The super‐sequence begins with HDQC, followed by SC‐CACO. This order cannot be reversed, as ^1^H decoupling during SC‐CACO would saturate the guanidinium protons of arginine, as well as water, thereby compromising the CP transfer. In HDQC, ^13^C pulses are selective for C_ζ_ to minimize perturbation of the C_γ_/C_β_ magnetization of Asp, Asn, Gln, and Glu residues. This experiment, based on the NOAH approach, exploits ^13^C detection and allows simultaneous investigation of the interactions involving positively charged arginine residues and negatively charged aspartate and glutamate residues (asparagine and glutamine are also detected). This method is based on the principle of using a single receiver. A second experiment is proposed to assign the resonances of the arginine's guanidinium group and exploits two different receivers, as for MR and UTOPIA type experiments. A scheme of the investigated amino acid side chains, with the relative nomenclature and chemical shift ranges in which the resonances are observed, is shown in Figure 1.

The approach, initially tested on ubiquitin, is demonstrated for the investigation of the electrostatic interaction between the N‐terminal domain of the SARS‐CoV‐2 nucleocapsid protein (N‐terminal domain [NTD]) and enoxaparin (EP), a negatively charged polysaccharide belonging to the glycosaminoglycan family. The NTD plays a key role in viral genome packaging and replication, and features several arginine residues distributed along flexible loop regions forming the so‐called basic finger, a structurally and functionally relevant motif for the interaction with negatively charged RNA backbones (Dinesh et al., 2020; Guseva et al., 2021; Pontoriero et al., 2022; Schiavina et al., 2021). Linear polyanions, such as EP, were recently proposed as possible ligands to interfere with the protein function (Bolognesi et al., 2025a; Bolognesi et al., 2025b; Schiavina et al., 2022; Tino et al., 2025). More importantly, the interaction between the N protein and heparan sulfate (a close analog of heparin) was recently shown as the driving force responsible for the presence of the virus on the surface of cells (infected as well as non‐infected neighboring ones) (Fahoum et al., 2025). This was in turn identified as a possible mechanism to stimulate an immune response by the host not only toward infected cells but also to healthy ones, exacerbating the effects of the COVID19 disease as well as of long COVID19 effects (Fahoum et al., 2025). The investigation of the interaction between EP and NTD of the SARS‐CoV‐2 nucleocapsid protein is thus of great importance to clarify the molecular features at the basis of the interaction and to stimulate further progress in the field.

By combining ^13^C direct detection with multiple acquisition strategies, we were able to observe and assign side chain resonances of positively charged arginine residues, including the terminal guanidinium groups that are often unobservable, in parallel to side chains of negatively charged residues, gaining new insights into the dynamic and electrostatic features of this biologically relevant interaction.

## 2 RESULTS AND DISCUSSION

### 2.1 Efficient design for the concurrent acquisition of two experiments

**FIGURE 2:**
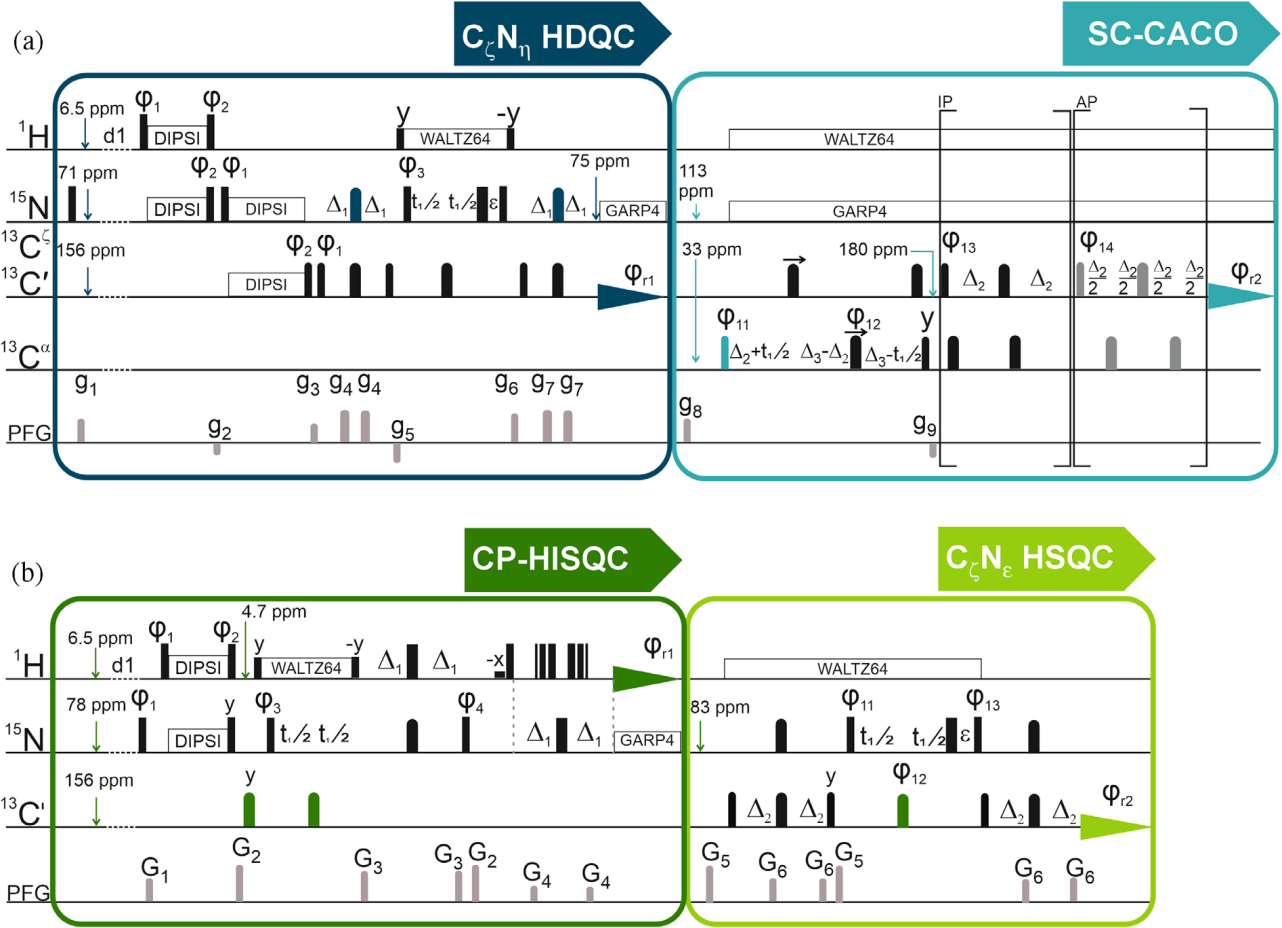
(a) The pulse sequence in which CP C_ζ_N_η_ HDQC and side‐chain‐selective SC‐CACO are combined. In the CP C_ζ_N_η_ HDQC part the Δ_1_ delay was set to 13.5 ms (1/2J_CζNη_). The phase cycle is: φ_1_ = *x* −*x*, φ_2_ = −*x x*, φ_3_ = 2(*x*) 2(*y*) 2(−*x*) 2(−*y*) and φ_r1_ = *x* −*x* −*x x*. Gradient strengths in G/cm are: g_1_ = 37.5, g_2_ = 16.5, g_3_ = 20.6, g_4_ = 5.9, g_5_ = 13.9, g_6_ = 29.6, g_7_ = 12.3. In the CACO part Δ_2_ is equal to 4.5 ms (1/4J_CαC′_) while Δ_3_ was set to 13.3 ms (1/2J_CC_). The phase cycle used is the following: φ_11_ = *x* −*x*, φ_12_ = 4(*x*) 4(*y*), φ_13_ = 2(*x*) 2(−*x*), φ_1_4 = 2(−*y*) 2(*y*) and φ_r2_ = *x* −*x* −*x x* −*x x x* −*x*. Gradients were applied with the following strengths in G/cm: g_8_ = 33, g_9_ = 12.4. A schematic representation of the MR H_ε_N_ε_//C_ζ_N_ε_ experiment is reported in (b), in which a N_ε/η_H_ε/η_ CP‐HISQC experiment is acquired during the relaxation delay of C_ζ_ magnetization. The phase cycle in the N_ε_H_ε_ CP‐HISQC part was achieved using: φ_1_ = *y* −*y*, φ_2_ = −*y y*, φ_3_ = 2(*y*) 2(−*y*), φ_4_ = 4(*x*) 4(−*x*) and φ_r1_ = *x* −*x* −*x x* −*x x x* −*x*. Gradient strengths in G/cm: G_1_ = 5.35, G_2_ = 10.7, G_3_ = 20.0, G_4_ = 12.8. Δ_1_ delays were set equal to 2.7 ms (1/4J_HN_), while Δ_2_ delays were set to 12.5 ms (1/4J_CζNε_). The phase cycle in the C_ζ_N_ε_ HSQC part was the following: φ_11_ = *x* −*x*, φ_12_ = 2(*x*) 2−(*x*), φ_13_ = 4(*x*) 4(−*x*), and φ_r2_ = *x* −*x x* −*x* −*x x* −*x x*. Gradients were set with the following strengths in G/cm: G_5_ = 32.1, G_6_ = 10.2. In all panels, narrow solid rectangles represent hard π/2 pulses, while wide solid rectangles represent hard π pulses; rounded shapes represent band‐selective shaped pulses which are accurately described in the Materials and Methods section (narrow and wide ones represent π/2 and π pulses, respectively). Quadrature detection was achieved through States‐TPPI by incrementing the phase of the π/2 pulse prior to the building block devoted to chemical shift evolution monitored in the indirect dimension.

The first experiment, shown in Figure 2a, was designed to simultaneously provide information about amino acid side chains with opposite charges, thereby offering a tool to study fine details of protein interfaces involved in electrostatic interactions. To this end the C_ζ_N_η_‐HDQC (Mackenzie & Hansen, 2017) was modified by including a double CP element (^1^H‐^15^N and ^15^N‐^13^C) at the beginning of the pulse sequence (CP C_ζ_N_η_‐HDQC). The initial CP step facilitates the magnetization transfer from exchangeable ^1^H_η/ε_ protons to ^15^N_η/ε_ nuclei, effectively exploiting the abundant water proton magnetization; the second one is used to transfer magnetization from ^15^N to ^13^C. This experiment also relies on the evolution of double quantum (DQ) coherence in the indirect dimension (^15^N_η_). DQ is less susceptible with respect to single quantum coherences to conformational exchange processes between the ^15^N_η_ nuclear spins arising from rotation of the guanidinium group around the N_ε_‐C_ζ_ axis (Nieto et al., 1997). This experiment was combined with a tailored version of the CACO (Bermel et al., 2005; Bermel et al., 2006; Pontoriero et al., 2020) (SC‐CACO) focusing on carbonamide and carboxylic groups of Asp, Asn, Glu, and Gln to access information about both positively (Arg) as well as negatively (Asp, Glu) charged side chains. We chose the CACO variant which exploits a constant‐time scheme to properly refocus *J*_CαCβ_ and *J*_CβCγ_ couplings (*J*_CC_) while transferring the magnetization from ^13^C_β_ to ^13^C_γ_ (Asp) and ^13^C_γ_ to ^13^C_δ_ (Glu) (Pontoriero et al., 2020). In this setup, the magnetization remains in the transverse plane for a period equal to 1/(*J*_CC_). This pulse sequence ensures proper refocusing of one‐bond ^13^C–^13^C couplings between aliphatic ^13^C nuclear spins. Selectivity on side chains is achieved using a π/2 selective pulse on C_β_ (asparagine/aspartate) and C_γ_ (glutamine/glutamate). Notably, glycine ^13^C_α_–^13^C′ resonances are also visible in this experiment, but their signal phase is opposite to the other peaks, making them easy to distinguish. The two magnetization‐transfer pathways are independent of each other and allow subsequent acquisition of individual experiments without sensitivity penalties (Figure S4, Supporting Information), while exploiting the same longitudinal relaxation delay. This reduces the overall experimental time and enables the simultaneous acquisition of different “snapshots.” Inspired by the NOAH approach (Kupče & Claridge, 2017; Yong et al., 2022) the C_ζ_N_η_‐HDQC//SC‐CACO experiment is thus designed to acquire ^13^C magnetization.

To obtain more detailed information on arginine side chains, the pulse sequence shown in Figure 2b was designed. This experiment is specifically tailored for the observation of arginine side chains and is based on the MR approach, combining CP‐HISQC (Yuwen & Skrynnikov, 2014) (^1^H‐detected) and C_ζ_N_ε_ HSQC (Werbeck et al., 2013) (^13^C detected) experiments. The resulting experiment was named MR H_ε/η_N_ε/η_//C_ζ_N_ε_. This MR strategy relies on the UTOPIA approach (Schiavina et al., 2019; Viegas et al., 2016). Here we exploit the longer longitudinal relaxation time of ^13^C nuclei with respect to ^1^H ones to acquire the H_ε/η_ N_ε/η_ experiment for free during the recovery delay of ^13^C_ζ_. In the CP‐HISQC, the magnetization is transferred from H_ε/η_ to N_ε/η_ via J‐CP transfer, followed by evolution of the N_ε/η_ resonances during which ^1^H decoupling scheme is applied to prevent effect of signal broadening and thus disappearance arising from exchange with water protons (Iwahara et al., 2007). The magnetization is then transferred back via the INEPT block, and the water signal is suppressed using 3‐9‐19 WATERGATE scheme (Sklenar et al., 1993). It is also possible to transfer the magnetization back from ^15^N to ^1^H using J‐CP transfer to avoid signal losses caused by exchange with bulk solvent. However, this approach can be very demanding in terms of the energy deposited into the probe, and it does not offer the advantage of exploiting water protons to enhance sensitivity (Yuwen & Skrynnikov, 2014). In the C_ζ_N_ε_ HSQC experiment, the starting magnetization source resides on ^13^C_ζ_ nuclear spins. The magnetization is then transferred to N_ε_, which is evolved in the indirect dimension and then transferred back to ^13^C_ζ_ for detection, both transfer steps use a regular INEPT scheme (Morris & Freeman, 1979). Selectivity is essential in NMR experiments focused on side chains, as it ensures that only the relevant spectral region is excited. In the case of the arginine guanidinium group, band‐selective pulses are crucial for ^15^N, as C^ζ^ is coupled to both N^ε^ and N^η^. The selective excitation of ^15^N^ε^ prevents unwanted multiple‐quantum coherences and ensures clean, interpretable spectra. It is worth nothing that ^1^H decoupling during ^13^C^ζ^ acquisition was omitted resulting in an optimal recovery of the water magnetization, needed for the following H_ε/η_ N_ε/η_ CP‐HISQC transient. Similar to the NOAH‐type approach described above, the UTOPIA approach allows the combination of orthogonal magnetization pathways without penalizing the sensitivity of the individual experiments (Figure S4).

### 2.2 Simultaneous snapshots of positive and negative side chain resonances to monitor interactions

The novel experiments have been used to study the NTD of the nucleocapsid protein from SARS‐CoV‐2 and its interaction with EP (Bolognesi et al., 2025b; Schiavina et al., 2022). NTD is characterized by the presence of 9 arginine residues and 11 acidic residues, including aspartic acid (Asp) and glutamic acid (Glu). Thus, it represents a suitable case of study for the proposed experiment that enables to directly monitor the key groups (positively charged guanidinium groups and negatively charged carboxylates) involved in electrostatic interactions.

**FIGURE 3:**
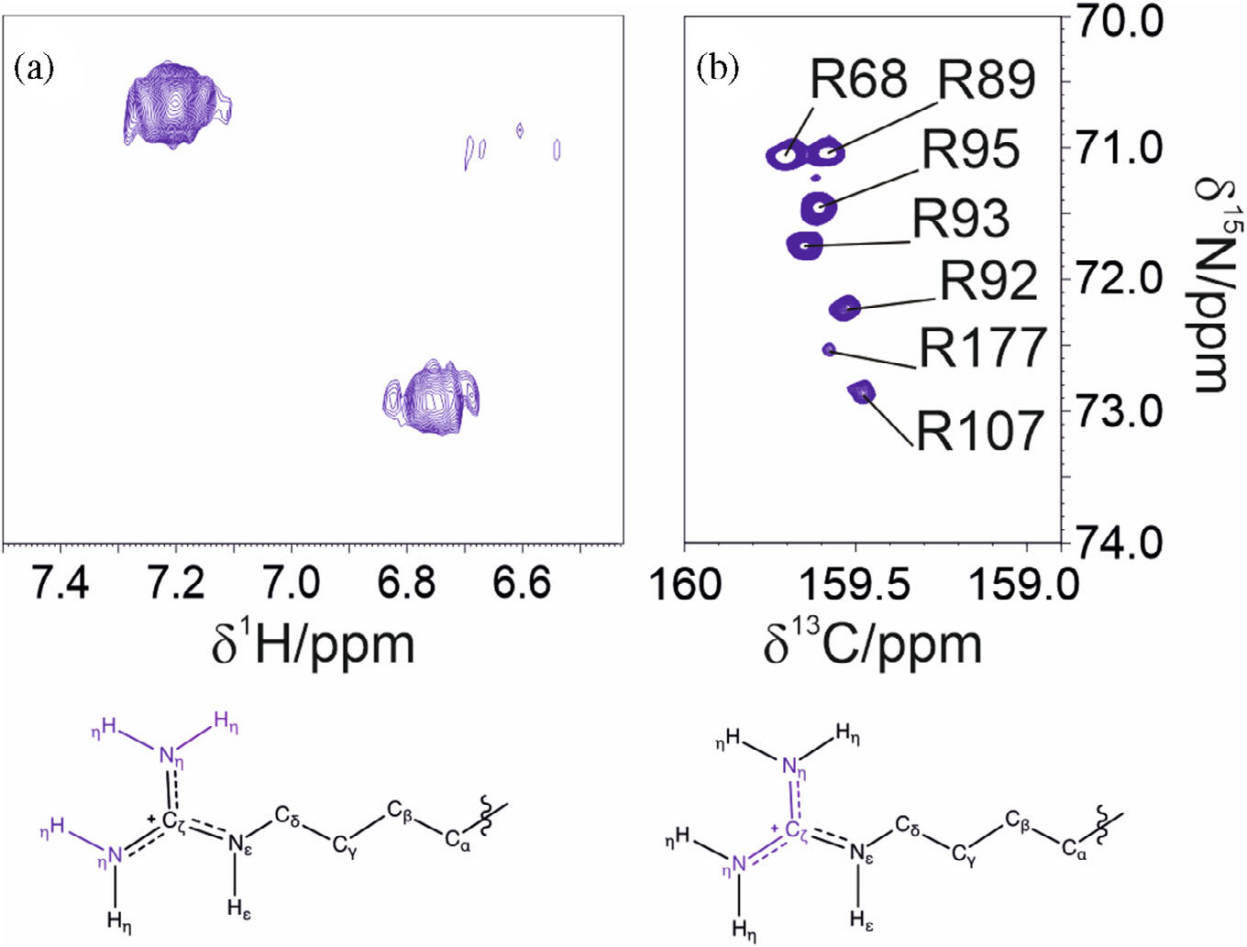
Comparison between ^1^H and ^13^C detected experiments. Panel (a) reports cross peaks corresponding to the H_η_–N_η_ correlation of arginine side chains acquired using ^1^H detection. Conformational and chemical exchange severely limit the observation of guanidinium resonances, resulting in significant line broadening. Panel (b) shows how ^13^C detection, combined with the use of double quantum coherence, mitigates this issue and enhances spectral resolution through the CP C_ζ_N_η_‐HDQC experiment. Worth noting, the evolution of the nitrogen chemical shift in the two spectra involves a single quantum coherence in the case of H_ε/η_N_ε/η_ spectrum in (a) and a double quantum coherence in the case of CP C_ζ_N_η_‐HDQC in (b). For this reason, the ^15^N_η_ chemical shift values in the two panels do not match exactly. The experiments were acquired on a 300 μΜ ΝTD sample.

Obtaining information on the ^15^N_η_ nuclear spins of the arginine guanidinium groups via conventional ^1^H‐detected methods is challenging due to severe line broadening caused by conformational and chemical exchange. As shown in Figure 3, ^13^C direct detection significantly improves signal detectability and spectral resolution by mitigating exchange‐induced broadening. This effect is further enhanced using DQ coherence, which facilitates observation of dynamic side chains through the CP C_ζ_N_η_‐HDQC experiment.

**FIGURE 4:**
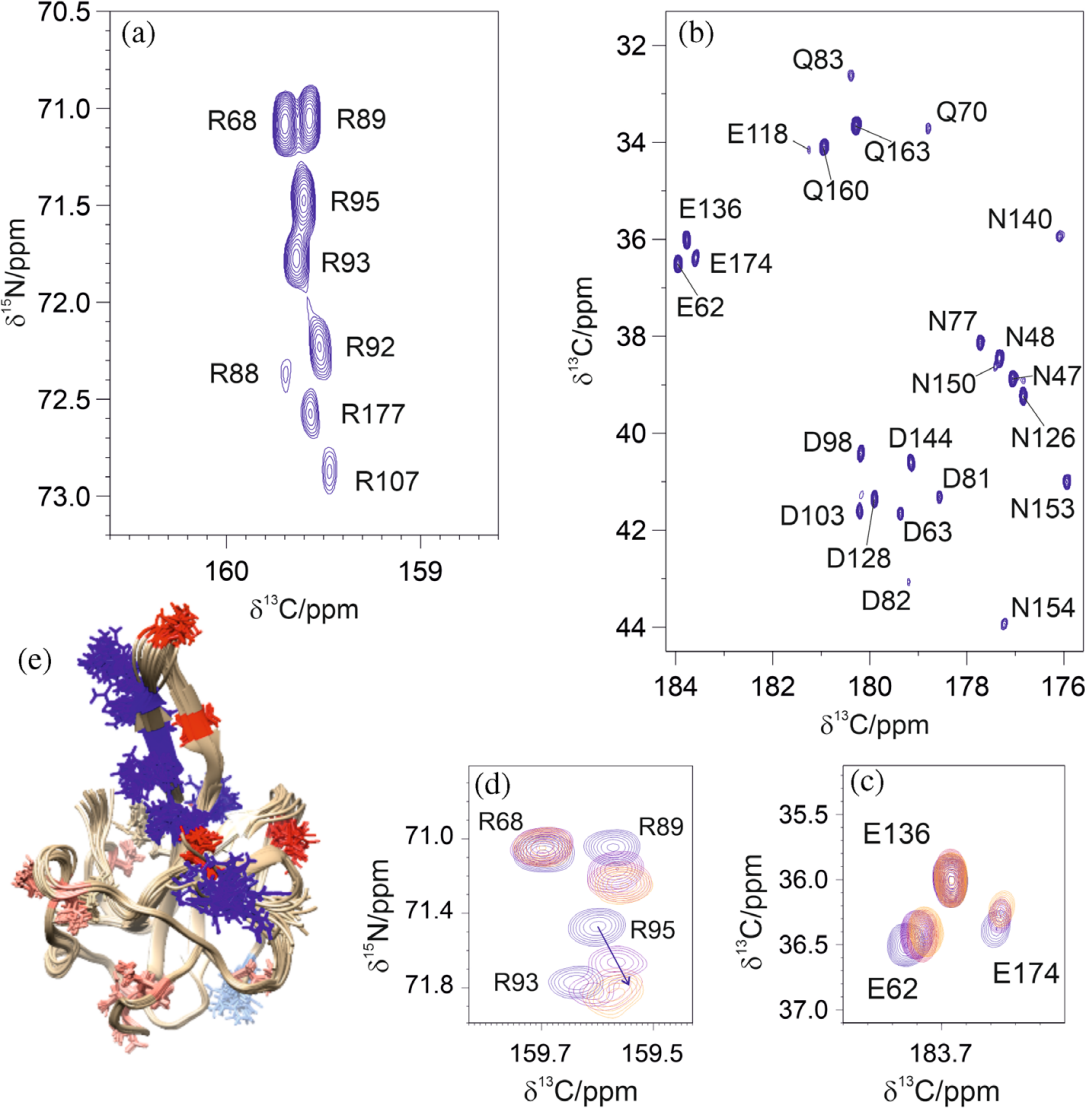
Titration of NTD with enoxaparin investigated using C_ζ_N_η_ HDQC//SC‐CACO experiments. Panel (a) shows the C_ζ_N_η_ HDQC spectrum and panel (b) shows the SC‐CACO spectrum of the free protein (without enoxaparin). The experiments were acquired on a 300 μΜ ΝTD sample. Panels (c) displays a zoom of the SC‐CACO spectrum, showing the perturbation of residues E62 and E174 upon addition of 0.6 equivalents (violet), 1.2 equivalents (red) and 2.4 equivalents (orange) of enoxaparin. E136 remains unaffected upon addition of enoxaparin. Panel (d) shows a zoom of the C_ζ_N_η_ HDQC spectrum illustrating the behavior of residues R68, R89, R93, and R95 upon addition of enoxaparin with the same color coding as in panel (c). Panel (e) shows a family of conformers (PDB 9QWI) where positively and negatively charged residues perturbed by the ligand are highlighted in vivid blue and red, respectively. Residues that remain unperturbed upon addition of enoxaparin are colored in pink (negative residues) and light blue (positive residues).

Based on these considerations, the guanidinium group of arginine residues and carboxylate groups of acidic residues were monitored using ^13^C‐detected NMR experiments through the NOAH‐based approach C_ζ_N_η_ HDQC//SC‐CACO (spectra reported in Figure 4a, b) upon interaction with increasing amounts of EP (spectra reported in Figure 4c, d). Sequence‐specific assignment of the cross peaks detected in the spectra involving arginine side chains was achieved exploiting also the complementary MR H_ε/η_N_ε/η_//C_ζ_N_ε_ experiment, as discussed in detail later in the text. Assignment of the carboxamide and carboxylic side chains of Asp, Asn, Glu, and Gln was achieved by adapting the available assignment (BMRB 51620) to the slightly different experimental conditions used in the present case (Bolognesi et al., 2025a).

In the C_ζ_N_η_ HDQC spectrum (Figure 4d), several arginine residues displayed chemical shift perturbations upon interaction with EP. Specifically, residues R89, R92, R93, and R95 showed significant perturbations. In contrast, R149 was not detected, likely due to its structural rigidity. Indeed, the analysis of the 20 conformers in the PDB entry 9QWI (Bolognesi et al., 2025a) indicates that the 9 arginine residues present in the protein construct (68, 88, 89, 92, 93, 95, 107, 149, and 177) exhibit different structural behaviors. Particularly, R88, R107, and R149 are found to establish a series of H‐bonds in all the available conformers thus resulting in a more rigid structure. On the other hand, the resonances arising from R68, R89, R92, R93, and R95 show intense peaks, correlated with the limited number of H‐bonds established by these residues in the deposited protein structure (Figure 4e). Interestingly, when EP is added, the backbone responds differently compared to the side chains. The side chain experiments provide further details on the interaction. Indeed, in these latter experiments we can observe more selective and more pronounced chemical shift perturbations with respect to what can be observed by monitoring the backbone signals (Figure S1). Even more, arginine residues R89, R93, R177, whose involvement in the interaction cannot be appreciated by looking at the interaction with the common backbone‐based strategies, are found to be highly perturbed exploiting the side chain‐based experiments.

The SC‐CACO experiment (Figure 4c) provides insights into the behavior of carboxamide and carboxylate moieties, such as those found in the side chains of aspartate, glutamate, asparagine, and glutamine residues, with the primary focus on the acidic side chains of aspartate and glutamate. The negative charge carried by these latter residues makes them unlikely to be the main interacting ones with the highly negative heparin‐based compound. However, the spectra revealed selective chemical shift perturbations for residues E62, D98, D103, D128, and E174. Among these, D98 and D103 are located in the basic finger region, while E62, D128, and E174 reside in loop regions adjacent to the basic finger. Moreover, D128 and E174 are involved in hydrogen bonding with residue R89 and R107, respectively. A second observed effect is the disappearance of peaks upon addition of EP. The disappearing peaks correspond to residues located in the most structured region of the protein (Figure 4e) and are likely to be broadened because of the increased rotational correlation time of the complex with respect to the free protein.

The complementarity of the two experiments acquired simultaneously highlights an elongated region rich in positively charged arginine residues involved in the interaction, confirming previously available information derived from backbone correlations and at the same time providing more direct information at the heart of the interaction as evidenced by the more pronounced and selective chemical shift changes (chemical shift perturbation values are reported in Figure S2). They also reveal the involvement of negatively charged residues which are likely to stabilize local conformations in the isolated protein that are disrupted when EP is added.

**FIGURE 5:**
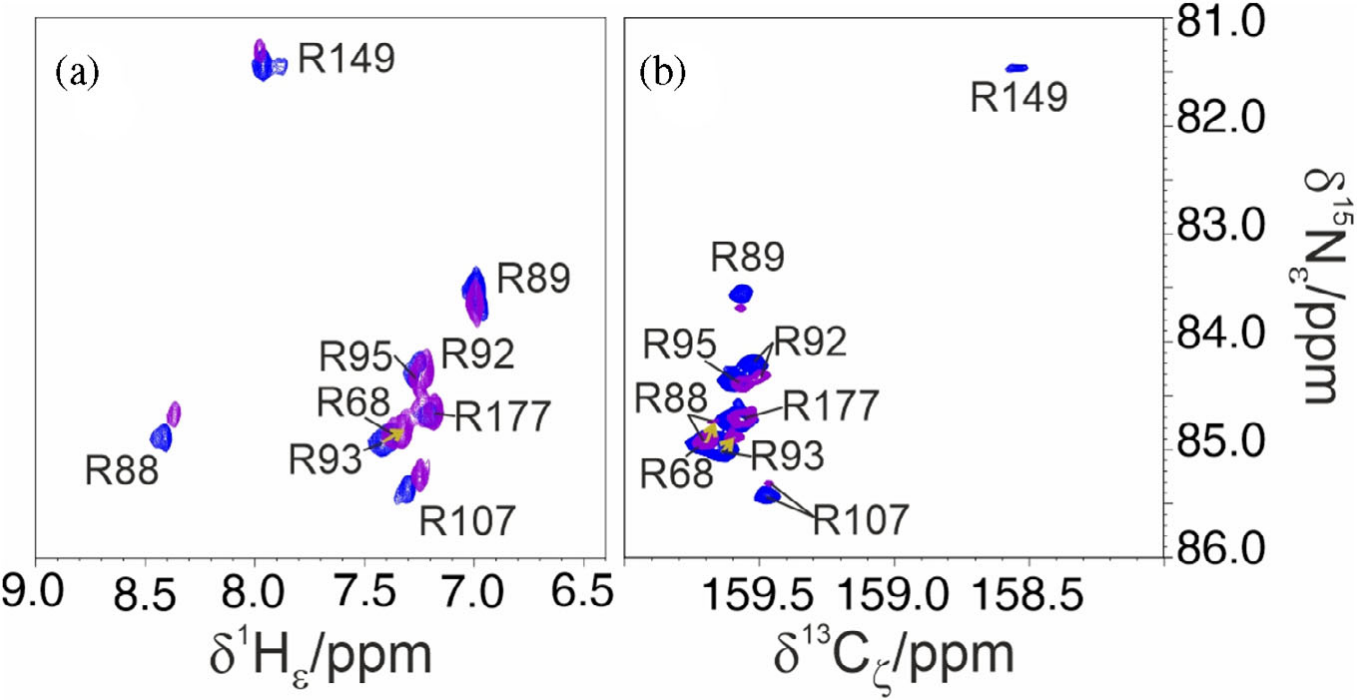
Titration of NTD with EP investigated using MR H_ε_N_ε_//C_ζ_N_ε_ experiments. The reference spectrum (without EP) is shown in blue, while the spectrum of NTD upon addition of 2.4 equivalents of EP is shown in violet. Panel (a) displays the H_ε_‐N_ε_ resonances, while panel (b) shows the corresponding C_ζ‐_N_ε_ cross‐peaks. Experiments were acquired on a 300 μΜ ΝTD sample.

To gain further insights into the interaction of the guanidinium of arginine residues upon interaction with EP, the MR H_ε_N_ε_//C_ζ_N_ε_ experiment was crucial. Figure 5 shows a reference spectrum of NTD (blue) and in complex with 2.4 equivalents of EP (violet). The H_ε_N_ε_ spectrum revealed that residues R89, R93, and R107 experienced pronounced chemical shift perturbations upon ligand binding, consistent with the C_ζ_N_η_ HDQC//SC‐CACO results. The H_ε_N_ε_ experiment also reported perturbation for R88 and R149. Residues R88, R89, and R93 are located in the basic finger region, while R107 is part of the β3 strand at the junction with the basic finger, a portion of the main interaction region. In contrast, residues R68 and R177, located at the edges of the protein, were unaffected by EP addition.

The involvement of the same arginine residues was also observed through the simultaneously acquired C_ζ_N_ε_ HSQC experiment. In this experiment, the possibility to observe the C_ζ_ nuclear spins provides an additional perspective on the interaction showing further interesting contributions. As an example, residues R92 and R95 (Figure 4b) are found to be more perturbed in this experiment as similarly observed in the C_ζ_N_η_ experiment (Figures S1, S2). Moreover, the availability of both spectra also contributes to resolve possible accidental overlaps in the ^1^H detected experiment, such as for residues 68, 92, 93, and 95.

It is worth noting that the C_ζ_N_ε_ HSQC spectrum is not affected by possible solvent exchange resonance broadening, ensuring full applicability in a variety of different experimental conditions, such as approaching physiological pH and temperature values, as also shown for backbone CON experiments (Felli & Pierattelli, 2022; Gil et al., 2013).

**FIGURE 6:**
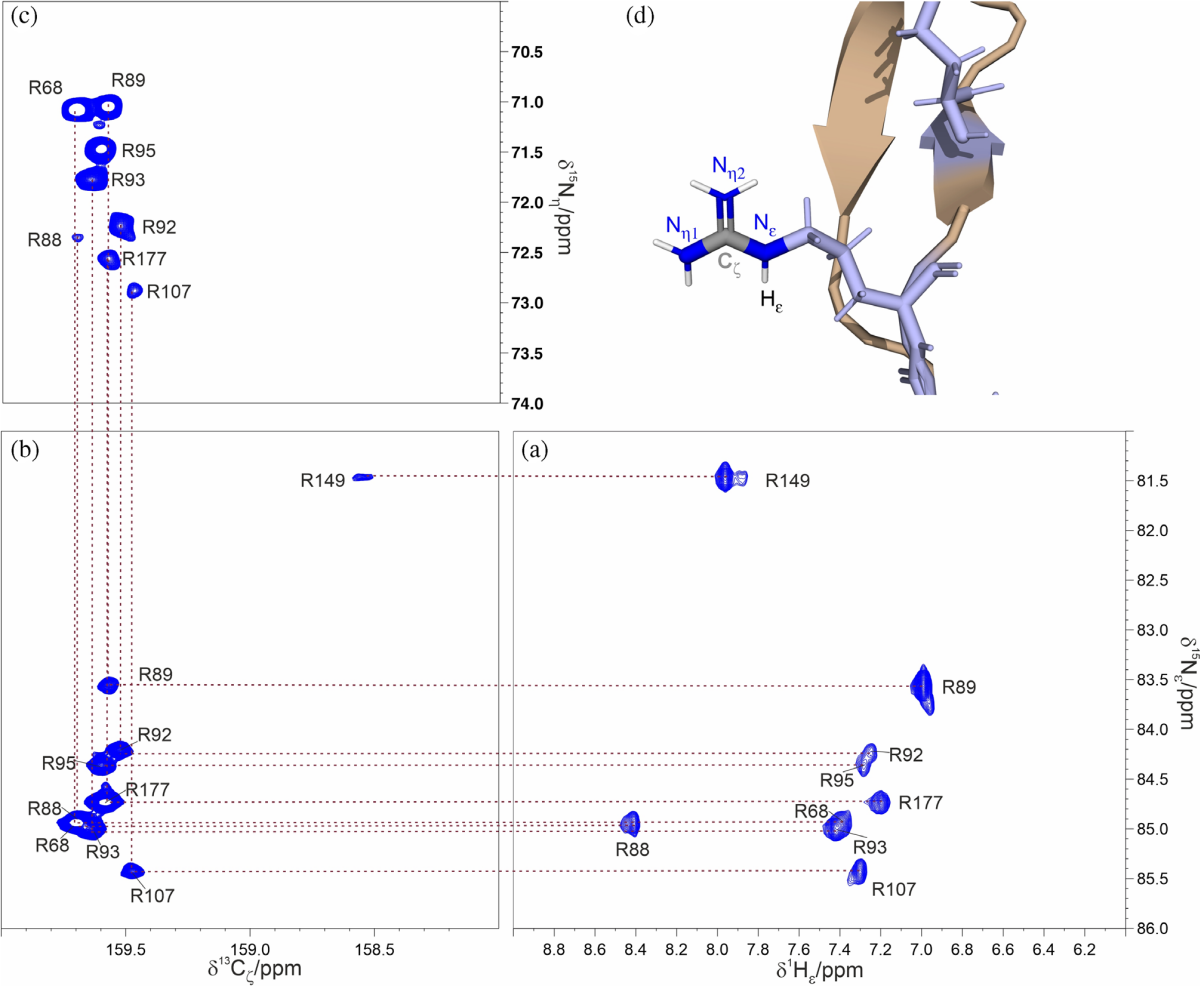
Panels (a–c) show the H_ε_N_ε_ CP‐HISQC, C_ζ_N_ε_ HSQC, and C_ζ_N_η_ HDQC spectra respectively, acquired with the novel pulse sequences described in this paper, demonstrating the ability to reconstruct resonance assignments across the entire guanidinium group. Experiments were acquired on a 300 μΜ ΝTD sample. Panel (d) reports a zoom of a conformer from PDB entry 9QWI illustrating the side chain of residue R93, together with standard nomenclature for each atom.

Combining the information available from the two proposed experiments with the previously available assignment for the H_ε_N_ε_ resonances (Schiavina et al., 2022), it is possible to assign all the resonances of the nuclear spins within the guanidinium group. Starting from the H_ε_N_ε_ resonances one can obtain the proper assignment of the C_ζ_ nuclei by observing the same N_ε_ chemical shift in the C_ζ_N_ε_ spectrum, as illustrated from the dashed lines reported from Figure 6a to Figure 6b. From this latter experiment one can in the end obtain the information about the N_η_ nuclear spins, as shown in Figure 6b, c, moving from the C_ζ_N_ε_ spectrum to the C_ζ_N_η_ one. Following the described procedure we fully assigned 8 out of 9 arginine side chains (H_ε_–N_ε_–C_ζ_–N_η_), with only a single arginine missing the N_η_ resonance, enabling us to retrieve nearly complete information for the terminal functional group of arginine residues in the NTD protein.

A comment is due on the general applicability of the proposed NMR approach and on possible limitations. The combined acquisition of several simultaneous NMR experiments enables access to independent parameters under identical sample conditions within a shorter experimental time. However, it is worth emphasizing that only experiments exploiting orthogonal magnetization transfer pathways can be combined within NOAH‐ or MR‐type approaches without compromising sensitivity. The overall experimental sensitivity is ultimately limited by the least sensitive experiment and otherwise matches that of the corresponding traditional experiments; it depends on multiple factors, including protein concentration, instrumental sensitivity, and the structural and dynamic properties of the investigated protein, making it difficult to define general guidelines. Nevertheless, the present results demonstrate that experiments applied to protein samples in the hundreds of micromolar range provide meaningful results. The heterogeneous structural and dynamic properties of the NTD, characterized by a well‐structured protein core as well as by a more flexible and disordered region, offer the opportunity to assess how experimental outcomes are modulated by the different dynamic properties of the protein (Bolognesi et al., 2025a; Dinesh et al., 2020; Schiavina et al., 2022). The results show that relaxation losses occurring for side chains that are part of well‐structured protein regions significantly affect the sensitivity of each of the two experiments combined for simultaneous acquisition, just like they would when acquiring the two experiments separately. In the presence of flexibility, as it occurs for solvent exposed side chains as well as for flexible protein loops, the experiments provide information that is difficult to access otherwise, as shown for the terminal part of the guanidinium group (N_η_) of arginine residues as well as for carboxylate groups of aspartate and glutamate residues. These are often key amino acids involved in interactions and are difficult to characterize at atomic resolution using other techniques, despite their key role in molecular recognition, signaling and function.

## 3 CONCLUSIONS

In conclusion, the experiments proposed to investigate the interaction between NTD from SARS‐CoV‐2 and EP enabled the direct observation of functional groups involved in electrostatic interactions, providing enhanced selectivity and sensitivity to binding‐induced changes compared with conventional approaches focusing on backbone nuclear spins. Notably, residues within the so‐called basic finger region of NTD exhibited the most pronounced perturbations in the presence of EP, reinforcing the pivotal role of this region in mediating the interaction. The ^13^C detected experiments acquired simultaneously through the NOAH‐based approach allowed us to capture the interaction by targeting both the guanidinium group of arginine residues, highlighting the key residues mediating the binding, and the carboxylate/carboxamide moieties of Asp, Asn, Glu and Gln. Among these latter residues, selective changes were detected in the negatively charged side chains within and adjacent to the primary region of the binding, suggesting that the interaction between negatively charged EP and the basic residues of NTD induces a rearrangement involving acidic residues, which also become perturbed. This description of the interaction is also supported by the presence of some residues whose peaks are not perturbed at all, which indeed belong to residues located in regions far from the main interaction site.

The combination of NOAH‐based and MR detection schemes, revealing information about different nuclear spins in a single experiment, opens the way to the design of a complete set of experiments to monitor key side chain resonances with the aim of identifying which are the main driving forces promoting the interaction of a target protein with possible partners. The novel experiments reveal information about highly flexible, solvent exposed residues thanks to the experimental design exploiting favorable features toward this objective. The focus on terminal functional groups of amino acids is particularly relevant to highlight key residues at the core of the interaction, information that is more difficult to access when investigating backbone nuclear spins which are generally sensitive to both direct and indirect effects.

The application of the proposed combined experiments can be extended and generalized to the study of various systems. The MR approach has already proven to be a useful tool for the study of several interactions (Knödlstorfer et al., 2024; Pontoriero et al., 2022; Schiavina et al., 2019) and can be further extended to study biomolecules where different nuclear spins can be observed. NOAH‐based experiments can instead be tailored to monitor other side chains such as those of aromatic residues, together with the backbone carbonyl carbon nuclei. The combined use of selective or orthogonal labeling strategies could open the way to the investigation of the primary actors driving interactions of proteins with cellular partners or complex ligands.

## 4 MATERIALS AND METHODS

All experiments were conducted at 298K by using a Bruker NMR spectrometer operating at 700.13 MHz ^1^H Larmor frequency, equipped with a cryogenically cooled probe‐head optimized for ^13^C direct detection (CP‐TXO).

The pulse sequences were tested on a sample of Ubiquitin (1.0 mM, 50 mM, sodium phosphate, pH 6.5) and the results are reported in Figure S3. The performances of pulse sequences were assessed by recording the experiment in the MR/NOAH approach and comparing the results with the 2D spectra acquired independently using the same acquisition parameters (number of scans, recovery delay, number of increments, acquisition time), reported in Figure S4.

The experiments were then applied to the NTD of the nucleocapsid (N) protein from SARS‐CoV‐2. The protein was expressed following the procedure reported in the literature (Schiavina et al., 2021), providing a sample with a final concentration of 300 μM in 25 mM potassium phosphate (K_3_PO_4_), 150 mM potassium chloride (KCl), 100 μM ethylenediaminetetraacetic acid (EDTA), 0.03% sodium azide (NaN₃), 5% deuterated water (D₂O), adjusted to pH 6.5.

For each pulse, the bandwidth (bw) reported corresponds to the frequency range over which the magnetization has dropped to 70.8% of its maximum value, as defined by the bandwidth factor in Bruker TopSpin's ShapeTool. The CP C_ζ_N_η_ HDQC experiment, inspired by the C_ζ_N_η_ HDQC one (Mackenzie & Hansen, 2017), was implemented together with SC‐CACO (Side Chains CACO) in a multiple acquisition NOAH‐based experiment. In the CP C_ζ_N_η_ HDQC experiment, the ^15^N Reburp pulse with 4.50 ms duration (bw 15 ppm) was centered in the middle of the ^15^N_η_ region (71 ppm); Q5 (for π/2) and Q3 (for π) band‐selective (Emsley & Bodenhausen, 1992) ^13^C pulses were centered at ^13^C_ζ_ (156 ppm) with a duration of 1.67 ms (bw 21 ppm) and 1.46 ms (bw 13 ppm), respectively. The ^1^H carrier was set to 6.5 ppm throughout the experiment. The experiment exploited ^1^H nuclear spins as the initial polarization source, with magnetization transfer to ^13^C_ζ_ achieved via double cross‐polarization (CP) transfer. The first CP step, from ^1^H to ^15^N, used a DIPSI (Shaka et al., 1988) scheme with a 9.5 ms contact time and a γB₁ field strength of 3.0 kHz. The second CP step, transferring magnetization from ^13^C to ^15^N, was performed using a DIPSI (Shaka et al., 1988) scheme with a 29 ms contact time and a γB₁ of 1.25 kHz. For the SC‐CACO experiment, a Q5‐shaped (Emsley & Bodenhausen, 1992) π/2 pulse with 1.8 ms duration (bw 19 ppm) was used to selectively irradiate the side‐chain resonances of aspartate, glutamate, asparagine, and glutamine. Other π and π/2 pulses were optimized for either the ^13^C^ali^ or ^13^C′ regions, using Q3 and Q5 shapes (Emsley & Bodenhausen, 1992) with duration 0.35 ms (bw 56 ppm) and 0.23 ms (bw 36 ppm), respectively. The ^13^C carrier was set at either 33 or 180 ppm.

The ^1^H‐^15^N CP‐HISQC (Yuwen & Skrynnikov, 2014), included in MR H_ε/η_N_ε/η_//C_ζ_N_ε_ experiment, was tailored to selectively irradiate ^15^N_ε/η_ of arginine residues side chains. To achieve this, ^15^N_ε/η_ resonances were irradiated using a Q3‐shaped (Emsley & Bodenhausen, 1992) selective π pulse with a 2.30 ms duration (bw 21 ppm), centered at 78 ppm. The ^1^H carrier was initially set to 6.5 ppm before ^1^H‐^15^N CP and later shifted to 4.7 ppm. ^1^H‐^15^N magnetization transfer was achieved using a CP‐DIPSI (Shaka et al., 1988) scheme, with a γB₁ of 3 kHz and a 9.5 ms contact time. In the C_ζ_N_ε_ HSQC (Werbeck et al., 2013) experiment, the ^13^C carrier was set at 156 ppm, while the ^15^N carrier was set at 83 ppm. Selective irradiation of the ^13^C region was achieved using Q3 and Q5 pulse shapes, with durations of 1.50 ms (bw 12 ppm) and 1.80 ms (bw 20 ppm), respectively. ^15^N selective pulses were designed to target N_ε_ resonances, using a Reburp (Geen & Freeman, 1991) shape with a 5 ms duration (bw 13 ppm). The ^13^C decoupling during the indirect evolution dimension was achieved using a Crp80, 0.5, 20.1 pulse with a 500 μs duration. ^1^H decoupling was performed using a 2.5 kHz WALTZ64 (Shaka et al., 1983; Zhou et al., 2007) supercycle, while ^15^N decoupling employed a 1.0 kHz Garp4 (Shaka et al., 1985) supercycle.

Protein‐ligand interaction was monitored using the above‐described NMR experiments. Reference spectra were recorded on an NMR sample containing the isolated protein (300 μM). Subsequent spectra were acquired on the same sample after the addition of controlled amounts of commercially available EP compound (CLEXANE, Sanofi), introduced through small aliquots of a 22 mM stock solution to reach NTD:EP molar ratios of 1:0.6, 1:1.2, 1:2.4.

All experiments were acquired and processed using TopSpin 4.4.0, and relevant processing details are reported in Data S1. Data analysis was conducted using CCPNMR v3.2.2 (Skinner et al., 2016).

All the pulse sequences exploited in this work are reported in the Bruker format at the end of Data S1.

## AUTHOR CONTRIBUTIONS

**Maria Anna Rodella:** Investigation; formal analysis; methodology; writing – original draft; writing – review and editing. **Marco Schiavina:** Methodology; investigation; formal analysis; writing – original draft; writing – review and editing. **Maksim Mayzel:** Methodology; validation; supervision; writing – review and editing. **Carlotta Cappanni:** Investigation; formal analysis. **Rainer Kümmerle:** Funding acquisition; supervision; writing – review and editing. **Roberta Pierattelli:** Conceptualization; methodology; funding acquisition; supervision; writing – original draft; writing – review and editing. **Isabella C. Felli:** Conceptualization; methodology; supervision; writing – original draft; writing – review and editing; funding acquisition.

## CONFLICT OF INTEREST STATEMENT

The authors declare no conflicts of interest.

## Supporting information


**Data S1:** Supporting information.

## Data Availability

The data that support the findings of this study are available from the corresponding author upon reasonable request.
