# Supplementary material for: At the core of the interaction: Probing charged side chains in flexible protein regions with simultaneous nuclear magnetic resonance experiments
Source: Protein Sci. 2026 Mar 13;35(4):e70533. doi: 10.1002/pro.70533 (PMC13140521; doi:10.1002/pro.70533)
Supplement: Supplementary file 1 — Data S1: Supporting information. [file PRO-35-e70533-s001.docx]

**Supplementary Information**

At the core of the interaction: probing charged side chains in flexible protein regions with simultaneous NMR experiments

Maria Anna Rodella^a#^, Marco Schiavina^a#^, Maksim Mayzel^b^, Carlotta Cappanni^a^, Rainer Kümmerle^b^, Roberta Pierattelli^a*^, and Isabella C. Felli ^a*^

a. Department of Chemistry “Ugo Schiff” and Magnetic Resonance Center (CERM), University of Florence, Florence, Italy

b. Bruker BioSpin AG, Fällanden, Switzerland

# The authors contributed equally to the work

Corresponding authors: *roberta.pierattelli@unifi.it, *isabellacaterina.felli@ unifi.it

[Figure SI1 2](#_Toc213405138)

[Figure SI2 3](#_Toc213405139)

[Figure SI3 4](#_Toc213405140)

[Figure SI4 5](#_Toc213405141)

[Processing details 6](#_Toc213405142)

[Figure SI5 7](#_Toc213405143)

[noah_splitcomb 8](#_Toc213405144)

[fixF1C.t1 10](#_Toc213405145)

[NOAH-based C_ζ_Nη HDQC//SC-CACO pulse sequence 12](#_Toc213405146)

[MR C_ζ_N_ε_//H_ε_N_ε_ pulse sequence 27](#_Toc213405147)


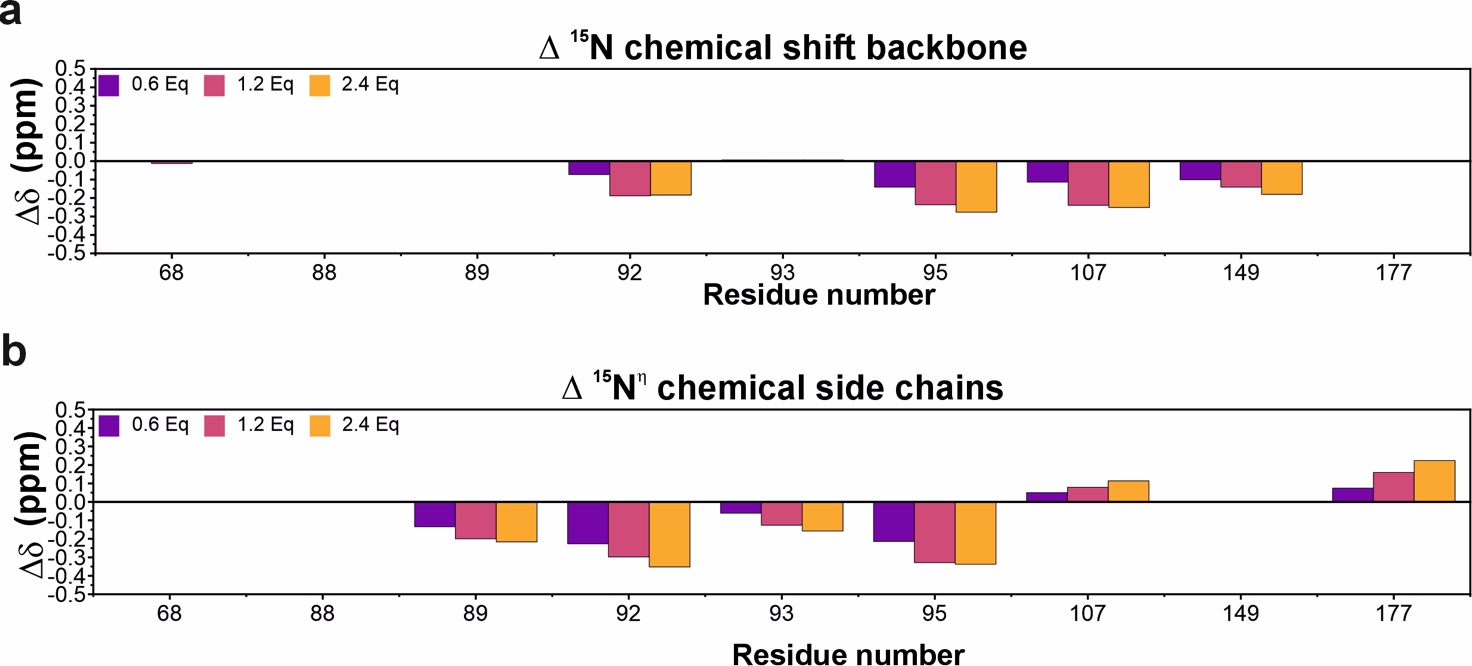


# Figure SI1

Panel **a** shows the chemical shift perturbations of backbone amide N^H^ groups observed in the ^1^H ^15^N HSQC spectra of the NTD upon addition of increasing equivalents of enoxaparin (0.6 eq, violet; 1.2 eq, pink; and 2.4 eq, orange). Panel **b** displays the N^η^ chemical shift differences measured in the C_ζ_N_η_ experiment under the same titration conditions. The side-chain experiment reveals that residues 89, 92, 93, and 95 are also perturbed upon addition of enoxaparin, providing complementary information. It should be noted that residues 149 and is not observed in the side-chain experiment while the resonance of residue 88 immediately disappears upon addition of enoxaparin.


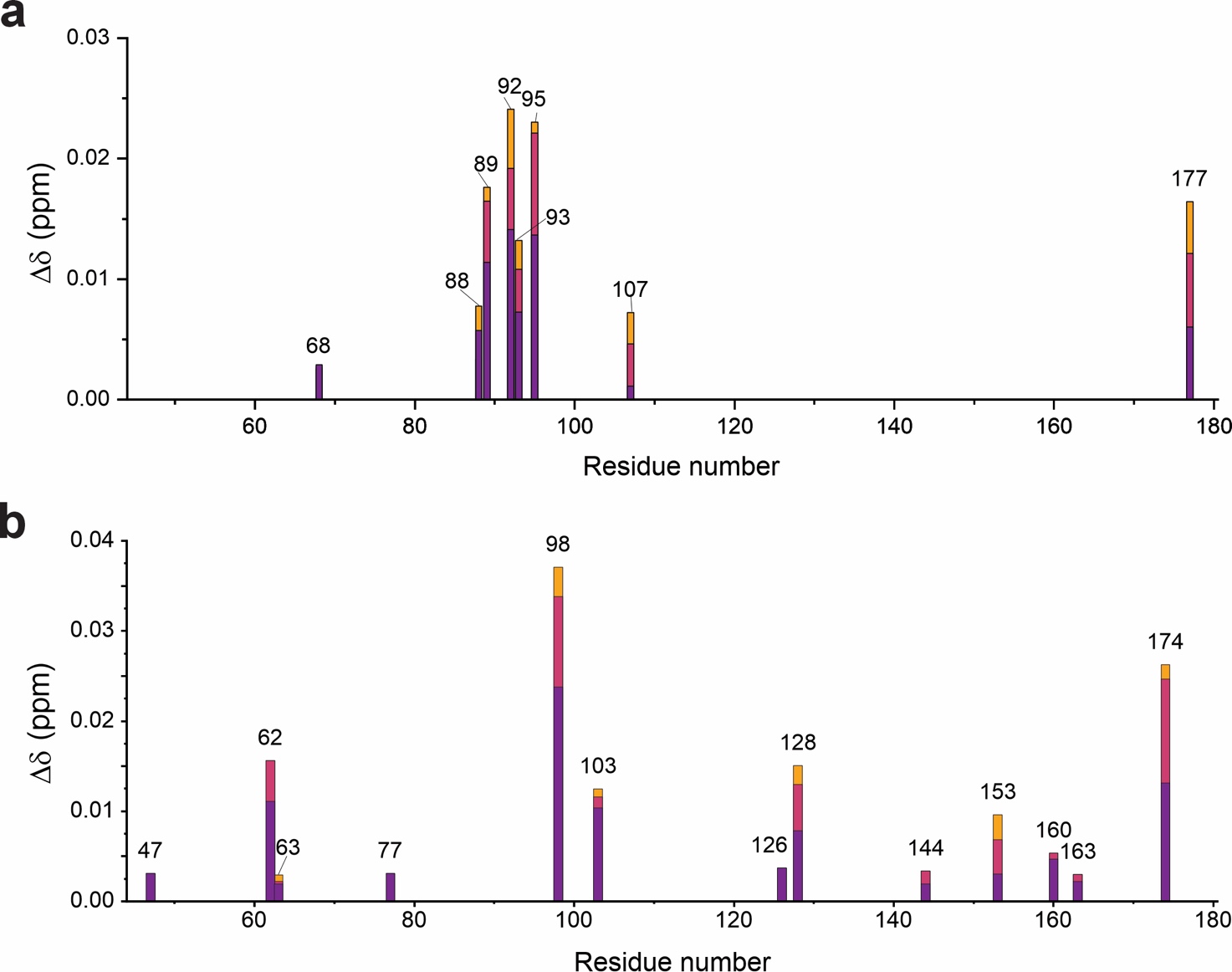


Figure SI2

Chemical shift perturbations of ^13^C^ζ^ and ^15^N^η^ nuclear spins detected in C_ζ_N_η_ HDQC (panel **a**) and C^β^,C^γ^ or C^γ^,C^δ^ nuclear spins detected in SC-CACO (panel **b**). These experiments were recorded on a sample of NTD upon addition of enoxaparin. The colour coding is the following: violet 0.6, pink 1.2, orange 2.4 equivalents of enoxaparin. The data reported in panel **a** confirmed that the interaction happens in the basic finger of NTD protein, where the higher CSP were found. It’s worth noting that some of the signals from SC-CACO experiment disappear (Q70, N75, D81, D82, Q83, E118, N140, N150, N154) or remain unperturbed (N47, N48, N77, N126, E136, D144, Q160, Q163) upon addition of enoxaparin and thus some of them are not visible in the plot reported here.


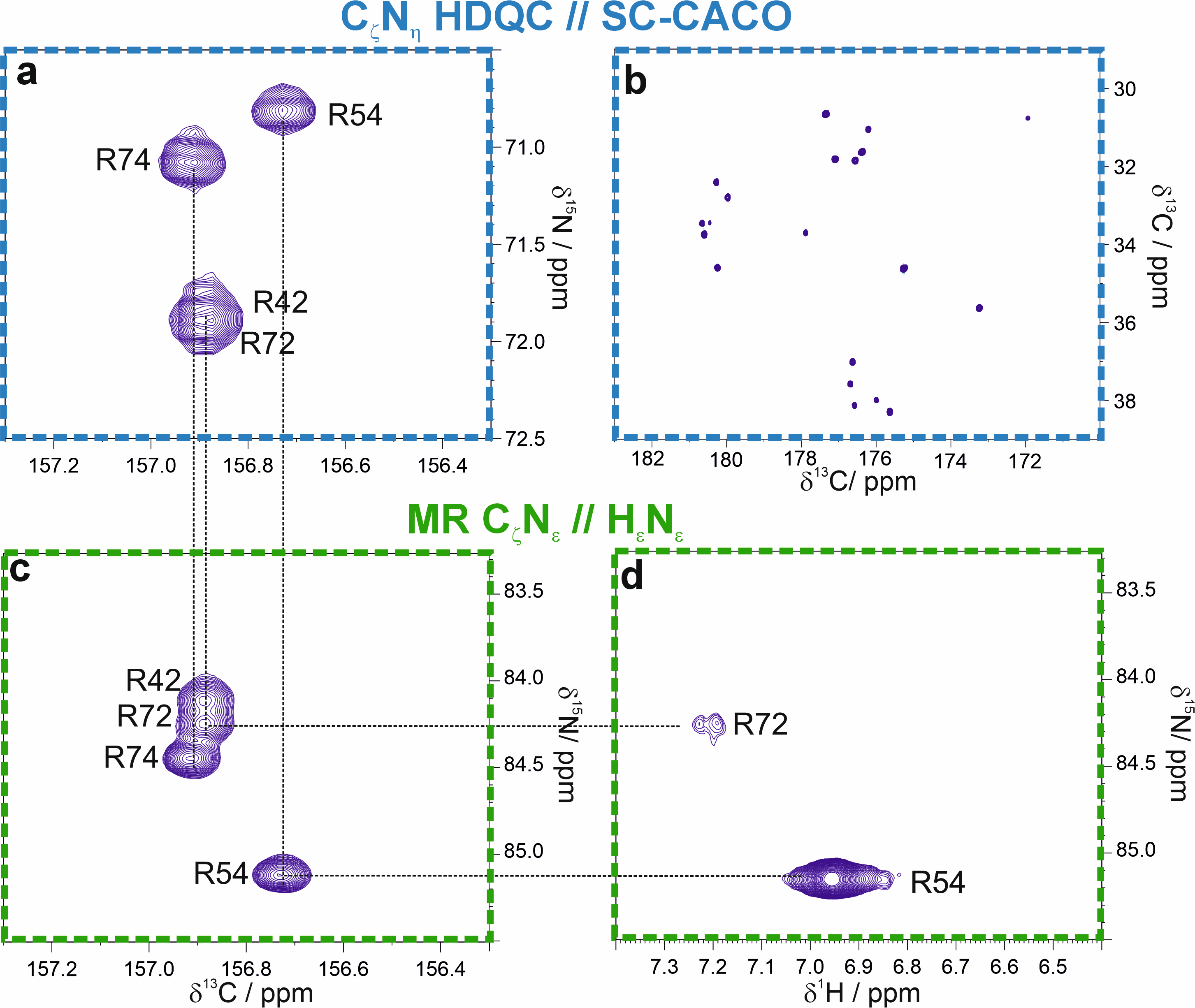


# Figure SI3

The figure shows the spectra acquired with the novel experiments: NOAH-based C_ζ_Nη HDQC//SC-CACO (panels **a** is the C_ζ_Nη HDQC experiment and **b** the SC-CACO, both framed in blue) and MR C_ζ_N_ε_/H_ε_N_ε_ (panels **c** represent the C_ζ_N_ε_ correlation while panel d is the H_ε_N_ε_ experiment, both framed in green). The novel experiments were tested on a uniformly labeled 1.0 mM Ubiquitin sample. The CP-HISQC spectrum (panel **d**) shows 2 out of 4 expected resonances from arginine side chains. In the other experiment tailored on Arginine side chains, C_ζ_Ν_ε_ HSQC (panel **c**) and C_ζ_N_η_ HDQC (panel **a**), 4 out of 4 arginine residues were detected. The assignment of the ^13^C-detected experiments was obtained using chemical shift values published by Yoshimura et al. (2016). The terminal functional group of of Asn, Asp, Gln, and Glu side chains were monitored using SC-CACO and the spectra is reported in panel **b**.


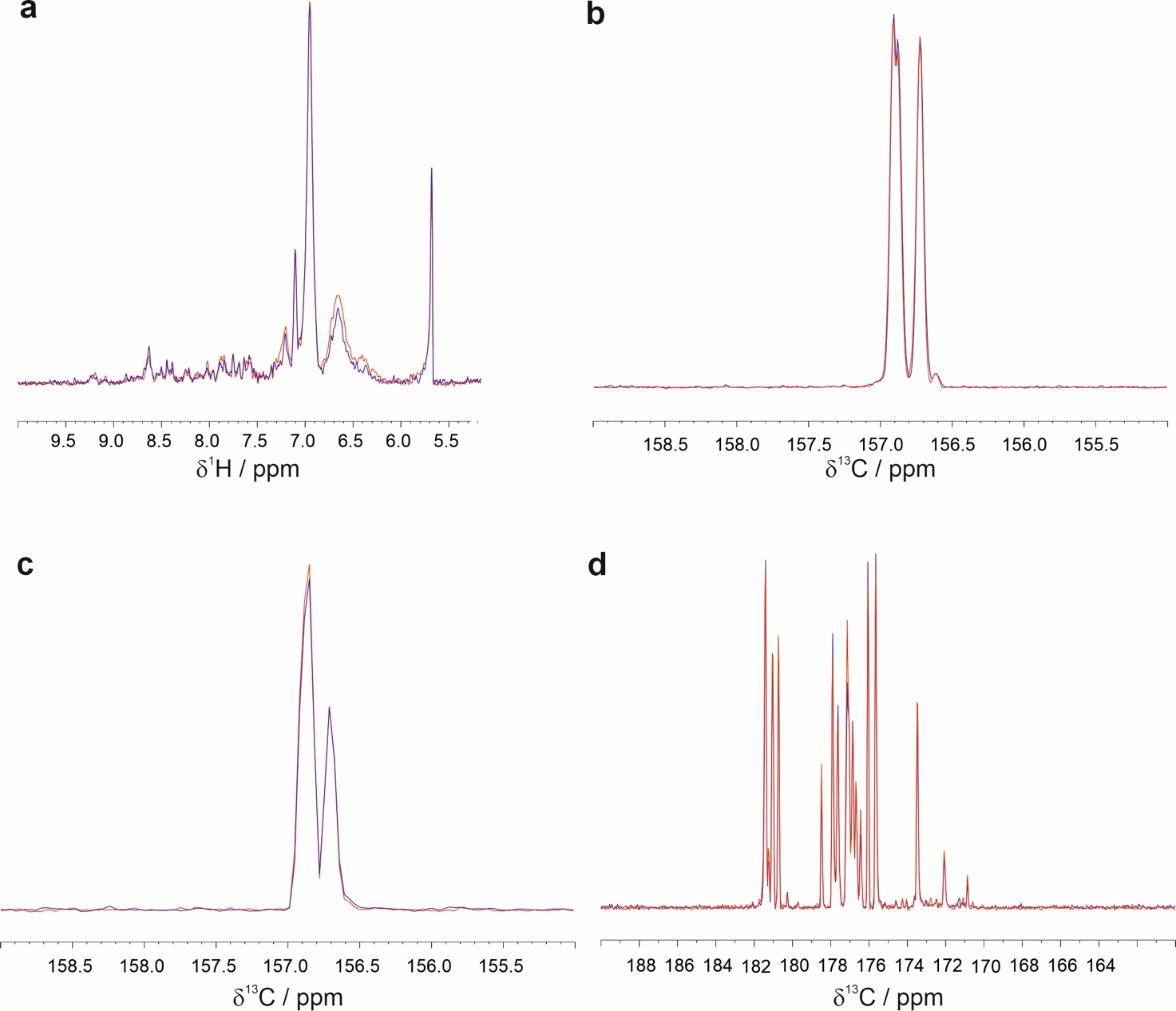


# Figure SI4

The horizonal positive projection of CP-HISQC (panel **a**) and HSQC C_ζ_Ν_ε_ (panel **b**) experiment show comparable results when combined using a Multiple Receiver approach (blue), and when recorded independently (red). The horizontal positive projection of the HDQC C_ζ_N_η_ (panel **c**) and SC-CACO (panel **d**) experiment, are also reported when acquired simultaneously through the NOAH-based experiment (in blue) and independently (in red). The simultaneous acquisition of the two spectra using this approach maintains the quality of the two experiments acquired separately (intensity variation not larger than 10%). The experiments were conducted on a 1.0 mM sample of Ubiquitin, uniformly labelled with ¹³C and ¹⁵N using a spectrometer operating at ^1^H Larmor frequency of 700 MHz.

# Processing details

The C_ζ_N_η_ HDQC//SC-CACO combination of experiments follows the same conceptual framework as the **NOAH** experiments, therefore, they share many of the processing strategies described on the website <https://nmr-genesis.co.uk>.

When multiple sub-experiments are acquired within a single dataset, data processing can be nontrivial. The following section outlines the main steps and the rationale for using specific processing scripts in the case of C_ζ_N_η_ HDQC//SC-CACO experiment.

In this scheme, the two different sub-experiments (HDQC and CACO) are acquired under the same experiment number. Consequently:

1. Within a single Bruker experiment, the free induction decays (FIDs) corresponding to the different sub-experiments (e.g., HDQC and CACO) are stored together.
2. In the CACO sub-experiment, two FIDs, corresponding to in-phase and anti-phase components are recorded in order to properly refocus the (J_CαC'_) coupling using a virtual homonuclear decoupling scheme. Evolution time in the indirect dimension of HDQC can be incremented either with every FID of CACO spectrum, resulting in a higher resolution spectrum, or with every second FID, meaning the same number of increments as in CACO but twice the number of scans. In our case, the limiting factor in HDQC was sensitivity rather than resolution and the later approach was used.
3. While it is only possible to define in TopSpin acquisition parameters for one sub-experiment, parameters for other sub-experiment of a super-sequence are typically defined via constants or hardcoded in the pulse sequence. In the case of HDQC/CACO super-sequence, F1 dimension defines ^15^N evolution in HDQC, and ^13^C evolution in CACO.

To address these issues, dedicated **AU programs** are required to separate and process the FIDs correctly. Specifically:

- The **splitx_au** program (available in the Bruker AU library) is used to split the FIDs of a single experiment into two independent datasets.
- For the HDQC sub-experiment, the two identical FIDs must be combined to yield a consistent dataset. This is achieved by specifying **noah_add** in the automation parameters, which sums the duplicate FIDs, effectively producing an experiment with twice the number of scans.
- The CACO sub-experiment, acquired using an IPAP scheme, must be processed with a procedure analogous to splitcomb here implemented as the noah_splitcomb AU program.
- Finally, the F1 dimension of the CACO dataset must be corrected to reflect the appropriate indirect nucleus ^13^C.

In practice once the processing AU program (AUNMP) entry is set to “splitx_au”, in Automation section and the user parameters are defined as:

**USERP1 = “noah_hsqc noah_aqq”** and **USERP2 = “noah_splitcomb**”,

the data can be processed by simply executing xaup.

A dialog window will appear asking to indicate the proper constant coupling for C^α^C’ (usually set to 53.8 Hz)

This procedure generates two new experiments, one with the suffix **##001** and another with **##003**, where **##** corresponds to the original experiment number. The dataset **##003** (CACO) must then be further processed using the AU program **fixF1C.t1** to correct the F1 nucleus assignment to ^13^C, a dialog window will appear indicating the offset, the

Please note that the au program noah_splitcomb and fixF1C.t1 (available in the following pages) are original and thus need to be added in the au program folder.


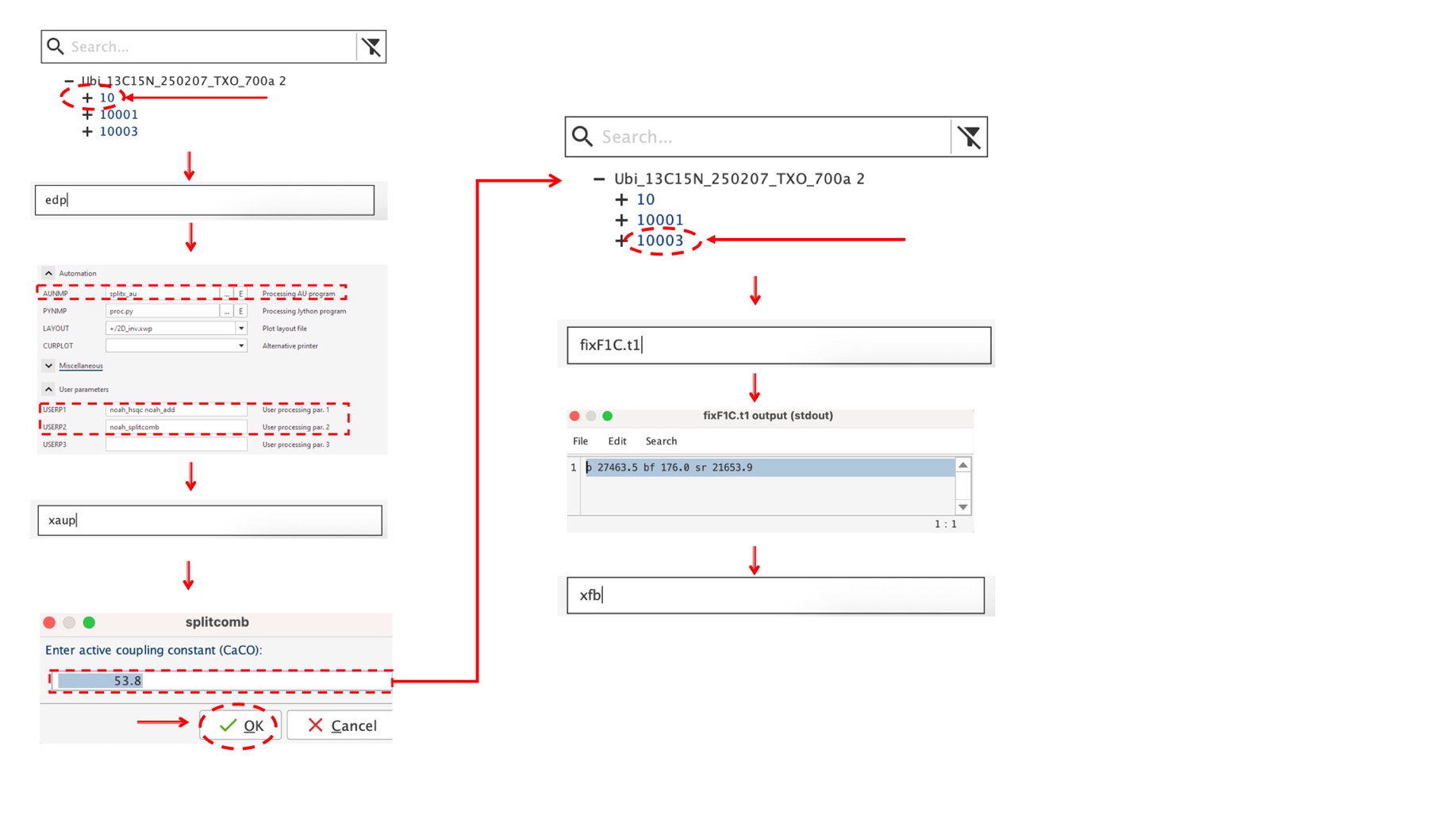


# Figure SI5

Flowchart of the processing procedure for C_ζ_N_η_ HDQC//SC-CACO experiments. In the reference experiment (##), go to the processing parameters section and enter the AU program and user processing parameters. Then run the xaup command to update the AU programs. Enter the desired J-coupling value. Next, go to the experiment ##003 and adjust the indirect dimension of the CACO experiment by running the fixF1C.t1 command from the command line. A window will open showing the offset (o), base frequency (bf), and spectrometer reference frequency (sr) values. Finally, process the data using the standard xfb command.

# noah_splitcomb

/*** ^^A -*-C++-*- **********************************************/

/* noah_splitcomb - NOAH IPAP exp that requires splitcomb */

/* 10.01.2025 */

/****************************************************************/

/* Short Description : */

/* AU program for processing IPAP experiment like C_CACO_IA*/

/****************************************************************/

/* Keywords : */

/* NOAH, noah_splitcomb */

/****************************************************************/

/* Description/Usage : noah_splitcomb iofs */

/* iofs is optional argument that may be used to offset */

/* expno for processed data, usually 1 or 2 */

/* Used in splitx_au. */

/****************************************************************/

/* Author(s) : */

/* Name : Maksim Mayzel */

/* Organisation : Bruker BioSpin */

/****************************************************************/

/* Name Date Modification: */

/* may 250110 created */

/****************************************************************/

/*

$Id:$

*/

char splitarg[64];

int oexpno = expno;

int nexpno = expno + 1;

if (i_argc > 2)

{

int i = atoi(i_argv[2]);

if (i > 0)

nexpno = expno + i;

}

XAU("fixF1C.t1","")

ERRORABORT

sprintf(splitarg, "ipap 2 %d y 1.0", nexpno);

XAU("splitcomb", splitarg)

ERRORABORT

DATASET(name, nexpno, procno, disk, user)

STOREPAR("AUNMP", "noah_hsqc")

Show_meta(SM_PROCP);

XAUPW

REFRESHDATA

DELETEEXPNO(name, oexpno, disk, user)

XCMD("sendgui browse_update_tree")

QUIT

# fixF1C.t1

/* fixF1C

* ------

* Fix the values of SW and O1 in the indirect dimension for NOAH 13C modules.

* SW is read from the status parameter cnst40.

* O3P is read from the status parameter cnst22.

*

* adapted version from fixF1n */

double sfo;

FETCHPARS("SFO1", &sfo)

STOREPAR1S("SFO1", sfo)

STOREPAR1("SFO1", sfo)

// cnst40 is 13C SW in ppm

float cnst40;

FETCHPAR("CNST 40", &cnst40)

STOREPAR1S("SW", (double) cnst40)

STOREPAR1("SW", (double) cnst40)

// calculate 13C SW_h

double sw_h;

sw_h = cnst40 * sfo;

STOREPAR1S("SW", cnst40)

STOREPAR1("SW", cnst40)

char nuc[8];

FETCHPARS("NUC1", nuc)

STOREPAR1S("NUC1", nuc)

STOREPAR1("NUC1", nuc)

double bf;

FETCHPARS("BF1", &bf)

STOREPAR1S("BF1", bf)

STOREPAR1("BF1", bf)

double o;

FETCHPARS("O1", &o)

STOREPAR1S("O1", o)

STOREPAR1S("O1", o)

float cnst22;

double sr;

FETCHPAR("CNST 22", &cnst22)

sr=o-cnst22*bf;

STOREPAR1("SR", sr)

printf("o %.1f bf %.1f sr %.1f",o,bf,sr);

QUIT

# NOAH-based C_ζ_Nη HDQC//SC-CACO

;noah2_cznhhdqc_caco.mar

;without selective pulses in HDQC

;cznhhdqc HDQC with CP H->N N->C

;cacp_ia

;

;$CLASS=HighRes

;$DIM=2D

;$TYPE=

;$SUBTYPE=

;$COMMENT=

prosol relations=<triple_c>

#include <Avance.incl>

#include <Delay.incl>

#include <Grad.incl>

define loopcounter ph1loop

"ph1loop=0"

"nbl=2"

"l0=1"

"l1=td1/(2*nbl)" ; real number of 1 TD =1 TD/ 2 (for IPAP) x nbl (as for NOAH)

"l2=l1/2" ; number of complex points = TD / 2 (for quadrature detection),

"d11=30m"

"d12=20u"

"p2=p1*2"

;"p4=p3*2"

"p22=p21*2"

"plw12=plw2*pow(p3/pcpd2,2)" ;f2 decoupling

"plw16=plw3*pow(p21/pcpd3,2)" ;f3 decoupling

; HDQC part

"d23=1s/(4*cnst1)"

;HN CP

"p6=1s/(4*cnst6)"

"plw22=plw2*pow(p3/p6,2)"

"plw23=plw3*pow(p21/p6,2)"

;NC CP

"p7=1s/(4*cnst7)"

"plw21=plw1*pow(p1/p7,2)"

"plw24=plw3*pow(p21/p7,2)"

"d6=l6*(p6*28.778)" ; define after p6 calculation

"d7=l7*(p7*28.778)" ; define after p7 calculation

;HDQC selective pulses 13C

"spnam13='Q5.1000'"

;"spnam13='Eburp2.1000'"

"p41=(bwfac13/(cnst13*bf1))*1e6"

"spw13=plw1*pow((p1*totrot13)/(p41*90*integfac13),2)"

"spoal13=1"

"spoffs13=0"

"spnam14='Q3.1000'"

"p42=(bwfac14/(cnst13*bf1))*1e6"

"spw14=plw1*pow((p1*totrot14)/(p42*90*integfac14),2)"

"spoal14=0.5"

"spoffs14=0"

"spnam15='Q5tr.1000'"

;"spnam15='Eburp2tr.1000'"

"spw15=plw1*pow((p1*totrot15)/(p41*90*integfac15),2)"

"spoal15=0"

"spoffs15=0"

;HDQC selective pulse 15N

"spnam32='Reburp.1000'"

"p54=(bwfac32/(cnst54*bf3))*1e6"

"spw32=plw3*pow((p21*totrot32)/(p54*90*integfac32),2)"

"spoal32=0.5"

"spoffs32=0"

"cnst26=0.5*(84+71)" ; 15N decoupling between Neta end Nepsilon

"in0=inf1/2"

"d0=2u"

"DELTA=d0*2+p42" ; define after p42 calc

"DELTA1=d23-p16-d16-(larger(p42,p54)/2)"

"DELTA2=d23-p16-d16-(larger(p42,p54)/2)-p41*cnst39"

;CACO Part

"spoffs26=bf1*((cnst21-cnst22)/1000000)"

"spoffs27=bf1*((cnst22-cnst21)/1000000)"

"spoffs28=0"

"o1_F1=bf1*cnst22/1000000"

; COCA pulses

"spnam23='Q5_sebop.1'"

"p11=(bwfac23/(cnst23*bf1))*1e6"

"spw23=plw1*pow((p1*totrot23)/(p11*90*integfac23),2)"

"spoal23=1"

"spoffs23=0"

"spnam24='Q3_surbop.1'"

"p12=(bwfac24/(cnst23*bf1))*1e6"

"spw24=plw1*pow((p1*totrot24)/(p12*90*integfac24),2)"

"spoal24=0.5"

"spoffs24=0"

"spnam25='Q5tr_sebop.1'"

"p25=(bwfac25/(cnst25*bf1))*1e6"

"spw25=plw1*pow((p1*totrot25)/(p25*90*integfac25),2)"

"spoal25=0"

"spoffs25=0"

"spnam26='Q3_surbop.1'"

"spw26=plw1*pow((p1*totrot26)/(p12*90*integfac26),2)"

"spoal26=0.5"

"spoffs26=bf1*((cnst21-cnst22)/1000000)"

"spnam27='Q3_surbop.1'"

"spw27=plw1*pow((p1*totrot27)/(p12*90*integfac27),2)"

"spoal27=0.5"

"spoffs27=bf1*((cnst22-cnst21)/1000000)"

"spnam28='Q5_sebop.1'"

;"p25=(bwfac28/(cnst25*bf1))*1e6"

"spw28=plw1*pow((p1*totrot28)/(p25*90*integfac28),2)"

"spoal28=1"

"spoffs28=0"

;CACO part

"d27=13.3m"

"d22=1s/(4*cnst2)"

"d10=d22+4u"

"d20=d27-p12-p12/2"

"DELTA11=d27-d22-p12-p12/2"

"in10=1e6/(2*cnst40*sfo1)"

"in20=in10"

1 ze

d11

2 d11 do:f2 do:f3

4u pl2:f2 pl3:f3 fq=0:f3

(p21 ph1):f3

p16:gp1

d16

4u BLKGRAD

3 d1

4u UNBLKGRAD

; HDQC

d12 st0

;H-N CP

(p3 ph21):f2 ;H -y/y

;begin DIPSI1

4u pl22:f2 pl23:f3 ; on Nh

4 (p6*3.556 ph23):f2 (p6*3.556 ph23):f3

(p6*4.556 ph25):f2 (p6*4.556 ph25):f3

(p6*3.222 ph23):f2 (p6*3.222 ph23):f3

(p6*3.167 ph25):f2 (p6*3.167 ph25):f3

(p6*0.333 ph23):f2 (p6*0.333 ph23):f3

(p6*2.722 ph25):f2 (p6*2.722 ph25):f3

(p6*4.167 ph23):f2 (p6*4.167 ph23):f3

(p6*2.944 ph25):f2 (p6*2.944 ph25):f3

(p6*4.111 ph23):f2 (p6*4.111 ph23):f3

lo to 4 times l6

;end DIPSI1

;Hz/N -y/y

2u pl2:f2 pl3:f3

(p21 ph22):f3 (p3 ph22):f2 ;Nz

p16:gp1*-0.44

d16

; N-C CP

(p21 ph21):f3 ; N -y/y

;begin DIPSI1

2u pl21:f1 pl24:f3 fq=cnst20(bf ppm):f1 ; on Cz

5 (p7*3.556 ph23):f1 (p7*3.556 ph23):f3

(p7*4.556 ph25):f1 (p7*4.556 ph25):f3

(p7*3.222 ph23):f1 (p7*3.222 ph23):f3

(p7*3.167 ph25):f1 (p7*3.167 ph25):f3

(p7*0.333 ph23):f1 (p7*0.333 ph23):f3

(p7*2.722 ph25):f1 (p7*2.722 ph25):f3

(p7*4.167 ph23):f1 (p7*4.167 ph23):f3

(p7*2.944 ph25):f1 (p7*2.944 ph25):f3

(p7*4.111 ph23):f1 (p7*4.111 ph23):f3

lo to 5 times l7

;end DIPSI1

;Nz/C -y/y

(p41:sp15 ph22) ;C y->z

p16:gp1*0.55

d16

;HDQC starts here

(p41:sp13 ph1)

DELTA1

p16:gp2

d16

(center (p42:sp14 ph0) (p54:sp32 ph0):f3 )

p16:gp2

d16

DELTA1

(p41:sp15 ph0)

p16:gp1*-0.37

d16 pl12:f2 pl3:f3

(p26 ph23):f2

4u cpd2:f2 ph0

(p21 ph2):f3

d0

(p42:sp14 ph0) ; having selective pulse here costs sensitivity

d0

(p22 ph0):f3

DELTA

(p21 ph0):f3

4u do:f2

(p26 ph25):f2

p16:gp1*0.79

d16

(p41:sp13 ph0)

DELTA2

p16:gp3

d16

(center (p42:sp14 ph0) (p54:sp32 ph0):f3 )

p16:gp3

d16 pl16:f3 fq=cnst26(bf ppm):f3 ; N between Nh and Ne

DELTA1

goscnp ph31 cpd3:f3 ;cpd3:f3 ; HDQC acquisition

4u do:f2 do:f3

;CACO

d12 st ; increment pointer to the next memory block

p16:gp1*0.88

d16 pl16:f3 pl12:f2

4u fq=cnst22(bf ppm):f1 fq=114(bf ppm):f3 ; Ca, NH

d12 cpd2:f2 cpd3:f3

(p25:sp28 ph13)

d10

(p12:sp26 ph11)

DELTA11

(p12:sp24 ph14)

d20

(p12:sp26 ph11)

4u

(p25:sp25 ph12)

p16:gp1*-0.33

d16 fq=cnst21(bf ppm):f1 ; CO

if "l0 %2 == 1"

{

(p11:sp23 ph15)

4u

(p12:sp27 ph11)

d22

(p12:sp24 ph11)

4u

(p12:sp27 ph11)

d22

}

else

{

(p11:sp23 ph16)

4u

d22*0.5

(p12:sp27 ph11)

d22*0.5

(p12:sp24 ph11)

4u

d22*0.5

(p12:sp27 ph11)

d22*0.5

}

go=2 ph30

4u do:f2 do:f3

30m wr #0 if #0 zd iu0

lo to 2 times 2 ; CACO ipap

"ph1loop+=1"

"phval2 = (ph1loop % 4) * 45" ; HDQC

4u ip2 + phval2

"phval13 = (ph1loop % 4) * 90"

4u ip13 + phval13

lo to 2 times 2

; HDQC t1-evolution

4u id0

4u ip2 + phval2

; CACO t1-evolution

4u id10

4u dd20

4u ip13 + phval13

lo to 2 times l2

4u BLKGRAD

d6

d7

exit

;HDQC

ph0=0

ph1=0 2

ph2=0 0 1 1 2 2 3 3

ph21=0 2

ph22=2 0

ph23=1

ph25=3

ph31=0 2 2 0

;CACO

ph11=0

ph12=1

ph13=0 2

ph14=0 0 0 0 1 1 1 1

ph15=0 0 2 2

ph16=3 3 1 1

ph30=0 2 2 0 2 0 0 2

;o1p: f1 channel - between Cz and CO [165 ppm]

;pl1 : f1 channel - power level for pulse (default)

;pl3 : f3 channel - power level for pulse (default)

;pl12: f2 channel - power level for CPD/BB decoupling

;pl16: f3 channel - power level for CPD/BB decoupling

;sp23: f1 channel - shaped pulse 90 degree (on resonance)

;sp24: f1 channel - shaped pulse 180 degree (on resonance)

;sp25: f1 channel - shaped pulse 90 degree (on resonance)

; for time reversed pulse

;sp26: f1 channel - shaped pulse 180 degree (C=O off resonance)

;sp27: f1 channel - shaped pulse 180 degree (Ca off resonance)

;sp28: f1 channel - shaped pulse 180 degree (Ca on resonance)

;p25: f1 channel - 180 degree shaped pulse (Ca, sp28)

;p8 : f1 channel - 180 degree shaped pulse for inversion (adiabatic)

;p11: f1 channel - 90 degree shaped pulse

;p12: f1 channel - 180 degree shaped pulse

;p16: homospoil/gradient pulse [1 msec]

;p21: f3 channel - 90 degree high power pulse

;p22: f3 channel - 180 degree high power pulse

;d0 : incremented delay (F1 in 2D) [3 usec]

;d1 : relaxation delay; 1-5 * T1

;l1 : Real 1 TD = 1 TD / (2 (IPAP) * NBL)

;l2 : Number of hyper complex points

;l6: Loop counter for CP transfer, set to adjust d6

;l7: Loop counter for CP transfer, set to adjust d7

;d6: H-N CP contact time [1/J=10ms for DIPSI, ~50-100ms for WURST]

;d7: N-C CP contact time [28ms]

;d11: delay for disk I/O [30 msec]

;d12: delay for power switching [20 usec]

;d16: delay for homospoil/gradient recovery

;d23: 1/(4J(NCO)) [12.5 msec]

;cnst1: Cz-Netta coupling in Hz [18-19]

;cnst2: CA-Co coupling in Hz [52]

;cnst6: HN CP (in Hz) [3125 Hz at 16.4T]

;cnst7: NC CP (in Hz) [1250 Hz at 16.4T]

;cnst10: scling factor SW(N)/SW(C)for CACO - not used

;cnst13: 13Cz selective pulses bandwidth (ppm) HDQC [15 ppm]

;cnst20: Cz chemical shift (offset, in ppm) [157 ppm]

;cnst21: C=O chemical shift (offset, in ppm) [180 ppm]

;cnst22: Ca chemical shift (offset, in ppm) [53 ppm]

;cnst23: 13C selective pulses bandwidth (ppm) CACO [86 ppm]

;cnst25: 13Ca selective pulses bandwidt (ppm) [25 ppm]

;cnst26: 15N offset for decoupling between Nz and Neta

;cnst39: chemical shift evolution during shaped 90-deg [Q5 -0.07, PC9 0.53, Eburp 0.69]

;cnst40: 13C Spectral window for indirect dimension (ppm)

;cnst54: 15N selective pulses bandwidth (ppm) [10-15 ppm]

;o1p: Cz chemical shift (156.75)

;inf1: 1/SW(N) = 2 * DW(N)

;in0: 1/(2 * SW(N)) = DW(N)

;nd0: 2

;ns: 8 * n

;ds: >= 32

;td1: number of experiments in F1

;FnMODE: States-TPPI (or TPPI) in F1

;cpd2: decoupling according to sequence defined by cpdprg2

;cpd3: decoupling according to sequence defined by cpdprg3

;pcpd2: f2 channel - 90 degree pulse for decoupling sequence

;pcpd3: f3 channel - 90 degree pulse for decoupling sequence

;use gradient ratio: gp 1

; 50

;for z-only gradients:

;gpz1: 70%

;gpz2: 11%

;gpz3: 23%

;gpz4: 31%

;gpz5: 13%

;gpz6: 28%

;gpz7: 12%

;use gradient files:

;gpnam1: SMSQ10.100

;gpnam2: SMSQ10.100

;gpnam3: SMSQ10.100

;gpnam4: SMSQ10.100

;gpnam5: SMSQ10.100

;gpnam6: SMSQ10.100

;gpnam7: SMSQ10.100

;$Id: $

# MR C_ζ_N_ε_//H_ε_N_ε_

;ut_cphsqc_czne.def

;avance-version (16/06/14)

;MR combining CP-HSQC and CzNe experiment

;HSQC

;2D H-1/X correlation via double inept transfer

;phase sensitive

;with decoupling during acquisition

;using f3 - channel

;water suppression using 3-9-19 pulse sequence with gradients

;

;T. Yuwen & N.R.Skrynnikov, J. Biomol. NMR, 58, 175-192 (2014)

;J. Lopez, R. Schneider, F.X. Cantrelle, I. Huvent & G. Lippens,

; Angew. Chem. Int. Ed. 55, x - x (2016)

;(G. Bodenhausen & D.J. Ruben, Chem. Phys. Lett. 69, 185 (1980))

;(M. Piotto, V. Saudek & V. Sklenar, J. Biomol. NMR 2, 661 - 666 (1992))

;(V. Sklenar, M. Piotto, R. Leppik & V. Saudek, J. Magn. Reson.,

; Series A 102, 241 -245 (1993))

;

;$CLASS=HighRes

;$DIM=2D

;$TYPE=

;$SUBTYPE=

;$COMMENT=

#include <Avance.incl>

#include <Grad.incl>

#include <Delay.incl>

;!!! not prosol compatible

"p2=p1*2"

"p22=p21*2"

"d11=30m"

"d12=20u"

"d13=4u"

;"d26=1s/(cnst4*4)" ;HSQC

"d23=12.5m" ;CzNe

"d0=3u" ;HSQC

"d32=3u" ;CzNe

"in0=inf1/2" ;HSQC

"in32=in2/2" ;CzNe

"DELTA3=d26-p16-d16-p27*2.385-d19*5-p1*2/PI" ;HSQC

"DELTA4=d26-p16-d16-p27*2.154-p0*0.231-d19*5-de-8u" ;HSQC

"DELTA11=d24-d0/2-p26/2-p8/2-p16-d16-4u-p44/2" ;HSQC

"DELTA12=d24+d0/2+p26/2+p8/2-p16-d16+2u-p44/2" ;HSQC

"DELTA13=d23-p16-d16-2u-larger(p14/2,p54/2)" ;CzNe

"DELTA15=d32*2+p8" ;CzNe ;CzNe

"d31=p32*28.778*l1" ;DIPSI duration

"l0=1" ;CEN

"l2=td1/2"

"spoffs2=0" ;CzNe ;CzNe

"spoffs3=0" ;CzNe ;CzNe ;CzNe

"spoffs8=0" ;CzNe ;CzNe

"spoffs13=bf2*(((cnst21-cnst26)*0.5)/1000000)" ;CzNe and HSQC

"acqt0=0"

;baseopt_echo

1 4u ze1

4u ze2

2 d11

3 d11

4 5m do:f3 do:f1

d1

4m pl16:f3

d12 pl1:f1

50u UNBLKGRAD

20u rpp26

(p21 pl3 ph13):f3

4u

p16:gp6

d16 fq=cnst19(bf ppm):f1 fq=cnst56(bf ppm):f3

(p1 pl1 ph13):f1

;begin DIPSI2:f1, DIPSI2:f3

5 (p32*3.556 pl41 ph20+ph26):f1 (p32*3.556 pl43 ph23+ph26):f3

(p32*4.556 pl41 ph21+ph26):f1 (p32*4.556 pl43 ph25+ph26):f3

(p32*3.222 pl41 ph20+ph26):f1 (p32*3.222 pl43 ph23+ph26):f3

(p32*3.167 pl41 ph21+ph26):f1 (p32*3.167 pl43 ph25+ph26):f3

(p32*0.333 pl41 ph20+ph26):f1 (p32*0.333 pl43 ph23+ph26):f3

(p32*2.722 pl41 ph21+ph26):f1 (p32*2.722 pl43 ph25+ph26):f3

(p32*4.167 pl41 ph20+ph26):f1 (p32*4.167 pl43 ph23+ph26):f3

(p32*2.944 pl41 ph21+ph26):f1 (p32*2.944 pl43 ph25+ph26):f3

(p32*4.111 pl41 ph20+ph26):f1 (p32*4.111 pl43 ph23+ph26):f3

0.050u ipp26

lo to 5 times l1

;end DIPSI2:f1, DIPSI2:f3

(p1 pl1 ph14):f1 (p21 pl3 ph12):f3

;4u fq=0:f3

p16:gp7

d16 fq=cnst18(bf ppm):f1

4u pl19:f1

(p26 ph12):f1

4u cpds1:f1 ph11

(p8:sp13 ph19):f2

(p21 pl3 ph15):f3

d0

(p8:sp13 ph11):f2

d0

4u do:f1

(p26 ph12):f1

2u

p16:gp8

d16

DELTA11 pl23:f3

(p2 pl1 ph12):f1 (p44:sp44 ph11):f3

DELTA12

p16:gp8

d16

(p21 pl3 ph16):f3

2u

p16:gp7

d16

(p11:sp1 ph19):f1

(p1 pl1 ph11):f1

DELTA3

p16:gp9

d16 pl18:f1

p27*0.231 ph17

d19*2

p27*0.692 ph17

d19*2

p27*1.462 ph17

DELTA

(p22 pl3 ph11):f3

DELTA

p27*1.462 ph18

d19*2

p27*0.692 ph18

d19*2

p0*0.231 ph18

4u

p16:gp9

d16

4u BLKGRAD

DELTA4 pl16:f3

goscnp1 ph31 cpds3:f3

4u do:f3

;czne

4u do:f3

50u UNBLKGRAD

p16:gp5

d16

20u pl19:f1 pl23:f3

20u fq=cnst26(bf ppm):f2

20u fq=cnst57(bf ppm):f3

d12 cpds1:f1

(p13:sp2 ph1):f2

DELTA13 ;d23

2u

p16:gp4

d16

(center (p14:sp3 ph1):f2 (p54:sp32 ph1):f3 )

DELTA13 ;d23

2u

p16:gp4

d16

(p13:sp8 ph2):f2

p16:gp5

d16 pl3:f3

4u ;cpds1:f1

(p21 ph3):f3

d32

(p8:sp13 ph5):f2

d32

(p22 ph1):f3

DELTA15

(p21 ph4):f3

4u do:f1

(p13:sp2 ph1):f2

DELTA13 pl23:f3 ;d23

2u

p16:gp4

d16

(center (p14:sp3 ph1):f2 (p54:sp32 ph1):f3)

DELTA13 ;d23

2u

p16:gp4

d16 pl16:f3 ;pl19:f1

4u BLKGRAD

go2=2 ph30 cpd3:f3 ;cpds1:f1

d11 do:f1 do:f3 wr1 #0 if1 #0 zd1

3m wr2 #1 if2 #1 zd2

;exp_f2 ipap

; 3m iu0

; lo to 2 times 2

;exp_f1 phase

3m ip16

;exp_f2 phase

3m ip3

lo to 3 times 2

;exp_f1 delay

3m id0

;exp_f2 delay

3m id32

lo to 4 times l2

d31

exit

ph1=0 ;CzNe

ph2=1 ;CzNe

ph3=0 2 ;CzNe

ph4=0 0 0 0 2 2 2 2 ;CzNe

ph5=0 0 2 2 ;CzNe

ph11=0 ;HSQC

ph12=1 ;HSQC

ph13=1 3 ;HSQC

ph14=3 1 ;HSQC

ph15=1 1 3 3 ;HSQC

ph16=0 0 0 0 2 2 2 2 ;HSQC

ph17=1 ;HSQC

ph18=3 ;HSQC

ph19=2 ;HSQC

ph20=0 ;HSQC DIPSI

ph21=2 ;HSQC DIPSI

ph23=0 ;HSQC DIPSI

ph25=2 ;HSQC DIPSI

ph26=0 2 2 0 ;HSQC DIPSI

ph31=0 2 2 0 2 0 0 2 ;HSQC

ph30=0 2 0 2 2 0 2 0 ;CzNe

;pl1 : f1 channel - power level for pulse (default)

;pl3 : f3 channel - power level for pulse (default)

;pl16: f3 channel - power level for CPD/BB decoupling

;pl18: f1 channel - power level for 3-9-19-pulse (watergate)

;pl19: f1 channel - power level for CPD decoupling

;pl41: f1 channel - power level for CP transfer (set accordingly to p32)

;pl43: f3 channel - power level for CP transfer (set accordingly to p32)

;p0 : f1 channel - 90 degree pulse at pl18

; use for fine adjustment

;p1 : f1 channel - 90 degree high power pulse

;p2 : f1 channel - 180 degree high power pulse

;p8 : f2 channel - 180 degree adiabatic (CzNe) [500 us at 700 MHz]

;p11: f1 channel - pulse on water [1 ms]

;p13: f2 channel - 90 degree shaped pulse (CzNe) [1.8 ms at 700 MHz]

;p14: f2 channel - 180 degree shaped pulse (CzNe) [1.5 ms at 700 MHz]

;p44: f3 channel - 180 degree selective pulse on N arg (HSQC) [2.3 ms at 700 MHz]

;p54: f3 channel - 180 degree selective pulse on Ne (CzNe) [5 ms at 700 MHz]

;p16: homospoil/gradient pulse

;p21: f3 channel - 90 degree high power pulse

;p22: f3 channel - 180 degree high power pulse

;p26: f1 channel - pulse for water allignment set as pcpd1

;p27: f1 channel - 90 degree pulse at pl18

;p28: f1 channel - trim pulse [1 msec]

;p32: f1 and f3 channel - pulse for CP transfer to cover around 3 kHz [83.3 us at 700 MHz]

;spnam1: Sinc1.1000

;spnam2: Q5_sebop.1

;spnam3:Q3_surbop.1

;spnam8: Q5tr_sebop.1

;spnam13: Crp80,0.5,20.1

;spnam32: Reburp.1000

;spnam44: Q3_surbop.1

;d0 : incremented delay (2D) [3 usec]

;d1 : relaxation delay; 1-5 * T1

;d11: delay for disk I/O [30 msec]

;d12: delay for power switching [20 usec]

;d13: short delay [4 usec]

;d16: delay for homospoil/gradient recovery

;d19: delay for binomial water suppression

; d19 = (1/(2*d)), d = distance of next null (in Hz)

;d26 : 1/(4J)YH

;d31 : DIPSI contact time (1/JNH)

;cnst4: = J(YH)

;cnst19 = 6.5 ppm (between H2O and HN)

;cnst18 = o1p = 4.7 ppm (H2O)

;cnst21 = 43 ppm (cali for decoupling)

;cnst26 = o2p = 156 ppm (Cz)

;cnst56 = o3p = 78 ppm (between Ne and Nh)

;cnst57 = 83 ppm (Ne)

;o1p = 4.7 ppm

;o2p = 156 ppm

;o3p = 78 ppm

;inf1: 1/SW(X) = 2 * DW(X)

;in0: 1/(2 * SW(X)) = DW(X)

;nd0: 2

;ns: 4 * n

;ds: 16

;td1: number of experiments

;FnMODE: States-TPPI, TPPI, States or QSEQ

;cpds1: decoupling according to sequence defined by cpdprg3: dipsi2

;cpd3: decoupling according to sequence defined by cpdprg3

;pcpd3: f3 channel - 90 degree pulse for decoupling sequence

;use gradient ratio: gp 1 : gp 2 : gp 3

; 80 : -50 : 30

;for z-only gradients:

;gpz4: 19%

;gpz5: 60%

;gpz6: 10%

;gpz7: 20%

;gpz8: 37.5%

;gpz9: 22%

;use gradient files:

;gpnam1: SMSQ10.100

;gpnam2: SMSQ10.100

;gpnam3: SMSQ10.100

;gpnam4: SMSQ10.100

;gpnam5: SMSQ10.100

;gpnam6: SMSQ10.100

;gpnam7: SMSQ10.100

;gpnam8: SMSQ10.100

;gpnam9: SMSQ10.100

;$Id: hsqcf3gpph19,v 1.8 2012/01/31 17:49:26 ber Exp $
